# Primary and Secondary siRNAs in Geminivirus-induced Gene Silencing

**DOI:** 10.1371/journal.ppat.1002941

**Published:** 2012-09-27

**Authors:** Michael Aregger, Basanta K. Borah, Jonathan Seguin, Rajendran Rajeswaran, Ekaterina G. Gubaeva, Anna S. Zvereva, David Windels, Franck Vazquez, Todd Blevins, Laurent Farinelli, Mikhail M. Pooggin

**Affiliations:** 1 Institute of Botany, University of Basel, Basel, Switzerland; 2 Fasteris SA, Plan-les-Ouates, Switzerland; 3 Biology Department, Indiana University, Bloomington, Indiana, United States of America; University of California Riverside, United States of America

## Abstract

In plants, RNA silencing-based antiviral defense is mediated by Dicer-like (DCL) proteins producing short interfering (si)RNAs. In *Arabidopsis* infected with the bipartite circular DNA geminivirus *Cabbage leaf curl virus* (CaLCuV), four distinct DCLs produce 21, 22 and 24 nt viral siRNAs. Using deep sequencing and blot hybridization, we found that viral siRNAs of each size-class densely cover the entire viral genome sequences in both polarities, but highly abundant siRNAs correspond primarily to the leftward and rightward transcription units. Double-stranded RNA precursors of viral siRNAs can potentially be generated by host RDR-dependent RNA polymerase (RDR). However, genetic evidence revealed that CaLCuV siRNA biogenesis does not require RDR1, RDR2, or RDR6. By contrast, CaLCuV derivatives engineered to target 30 nt sequences of a *GFP* transgene by primary viral siRNAs trigger RDR6-dependent production of secondary siRNAs. Viral siRNAs targeting upstream of the *GFP* stop codon induce secondary siRNAs almost exclusively from sequences downstream of the target site. Conversely, viral siRNAs targeting the *GFP* 3′-untranslated region (UTR) induce secondary siRNAs mostly upstream of the target site. RDR6-dependent siRNA production is not necessary for robust *GFP* silencing, except when viral siRNAs targeted *GFP* 5′-UTR. Furthermore, viral siRNAs targeting the transgene enhancer region cause *GFP* silencing without secondary siRNA production. We conclude that the majority of viral siRNAs accumulating during geminiviral infection are RDR1/2/6-independent primary siRNAs. Double-stranded RNA precursors of these siRNAs are likely generated by bidirectional readthrough transcription of circular viral DNA by RNA polymerase II. Unlike transgenic mRNA, geminiviral mRNAs appear to be poor templates for RDR-dependent production of secondary siRNAs.

## Author Summary

RNA silencing directed by small RNAs (sRNAs) regulates gene expression and mediates defense against invasive nucleic acids such as transposons, transgenes and viruses. In plants and some animals, RNA-dependent RNA polymerase (RDR) generates precursors of secondary sRNAs that reinforce silencing. Most plant mRNAs silenced by miRNAs or primary siRNAs do not spawn secondary siRNAs, suggesting that they may have evolved to be poor templates for RDR. By contrast, silenced transgenes often produce RDR-dependent secondary siRNAs. Here we demonstrate that massive production of 21, 22 and 24 nt viral siRNAs in DNA geminivirus-infected *Arabidopsis* does not require the functional RDRs RDR1, RDR2, or RDR6. Deep sequencing analysis indicates that dsRNA precursors of these primary viral siRNAs are likely generated by RNA polymerase II-mediated bidirectional readthrough transcription on the circular viral DNA. Primary viral siRNAs engineered to target a *GFP* transgene trigger robust, RDR6-dependent production of secondary siRNAs, indicating that geminivirus infection does not suppress RDR6 activity. We conclude that geminiviral mRNAs, which can potentially be cleaved by primary viral siRNAs, are resistant to RDR-dependent amplification of secondary siRNAs. We speculate that, like most plant mRNAs, geminiviral mRNAs may have evolved to evade RDR activity.

## Introduction

RNA silencing directed by miRNAs, short interfering (si)RNAs and PIWI-interacting RNAs is involved in regulation of gene expression and chromatin states and in defense against invasive nucleic acids such as transposons, transgenes and viruses [1]–[3]. Virus-infected plants accumulate high levels of viral siRNAs (vsRNAs) of three major size-classes: 21-nt, 22-nt and 24-nt [4], [5]. In *Arabidopsis thaliana* infected with DNA viruses, all four Dicer-like (DCL) enzymes are involved in processing of vsRNA duplexes from longer double-stranded RNA (dsRNA) precursors: DCL4 and DCL1 generate 21-nt class, DCL2 generates 22-nt class and DCL3 generates 24-nt class; 21-nt and 24-nt vsRNAs accumulate at higher levels than 22-nt vsRNAs [6]–[8]. By contrast, in RNA virus-infected *Arabidopsis*, DCL4-dependent 21-nt vsRNAs and/or DCL2-dependent 22-nt vsRNAs are the most abundant species, whereas DCL3-dependent 24-nt vsRNAs accumulate at much lower levels [7], [9], [10]. This reflects the difference in viral life cycles: DNA viruses transcribe their genomes in the nucleus, whereas RNA viruses are generally restricted to the cytoplasm. Likewise, plant endogenous genes and transgenes that undergo transcriptional silencing spawn predominantly DCL3-dependent 24-nt siRNAs, whereas those that undergo post-transcriptional silencing spawn predominantly DCL4-dependent 21-nt siRNAs and, in certain cases, DCL2-dependent siRNAs [1], [11], [12].

In endogenous and transgene-induced silencing pathways, dsRNA precursors of siRNAs can be generated by RNA-dependent RNA-polymerase (RDR). The *Arabidopsis thaliana* genome encodes six RDRs, three of which have been implicated in siRNA biogenesis [13]. RDR2 is required for biogenesis of 24-nt heterochromatic siRNAs (hcsiRNAs) mainly originating from repetitive DNA loci including transposons. RDR6 is required for biogenesis of *trans*-acting siRNAs (tasiRNAs), natural antisense transcript siRNAs and siRNAs derived from posttranscriptionally-silenced transgenes [1]. RDR6 is also involved in production of secondary siRNAs from some protein-coding genes targeted by miRNAs [14], [15]. RDR1 has so far been implicated in viral siRNA biogenesis (see below) and its function in endogenous or transgene-induced silencing is not known. Presumptive single-stranded RNA templates for RDR2 are produced by plant-specific RNA polymerases Pol IV and/or Pol V, but little is known about Pol IV and Pol V transcripts and RDR2-dependent dsRNAs [16]. dsRNA precursors of tasiRNAs originate from Pol II transcripts of *TAS* genes, which are cleaved by a miRNA::Argonaute (AGO) protein complex [17]–[20]. Either the 3′ cleavage product or the 5′ cleavage product is converted by RDR6 to dsRNA: RDR6 recruitment to only one of the two cleavage products is determined by 22-nt size of the initiator miRNA produced from a bulged hairpin precursor [21]–[23] or a second binding site of the miRNA::AGO complex [17], [19], respectively.

The possible role of RDRs in vsRNA biogenesis has been extensively studied using *A. thaliana* single, double and triple null mutants for RDR1, RDR2 and RDR6 [8], [24]–[28]. These studies produced rather conflicting results, but in many cases, wild type viruses were shown to predominantly spawn RDR-independent vsRNAs [29]. However, mutant RNA viruses with deletion or point mutation in the viral silencing suppressor gene spawn RDR6- and/or RDR1-dependent vsRNAs [26]–[28]. As a consequence the suppressor-deficient RNA viruses could establish systemic infection only on *A. thaliana* mutant plants lacking RDR6 and/or RDR1 activity. Nevertheless, suppressor-deficient RNA viruses spawn substantial amounts of RDR-independent vsRNAs. Thus, one of the major precursors of RNA virus-derived vsRNAs is likely a double-stranded replicative intermediate, transiently produced by viral RNA-dependent RNA-polymerase (vRdRP). Primary vsRNAs generated from such precursors may trigger RDR-dependent production of secondary siRNAs.

Plant DNA viruses do not encode a vRdRP. However, the biogenesis of DNA virus-derived vsRNAs does not appear to involve host RDRs. Thus, *Cauliflower mosaic virus* (CaMV)-derived vsRNAs of all major classes accumulate at comparable high levels in *A. thaliana* wild-type and *rdr1 rdr2 rdr6* triple mutant plants and their long dsRNA precursors are likely generated by Pol II [8]. The lack of RDR-dependent vsRNAs can be explained by the ability of a CaMV silencing suppressor protein to interfere with DCL4-mediated processing of dsRNAs produced by RDR6 [30], [31]. Silencing suppressor proteins of DNA geminiviruses have not been reported to interfere with RDR activity or DCL-mediated processing of RDR-dependent dsRNAs. In *A. thaliana* null mutants for Pol IV, RDR2, or RDR6 activity, the biogenesis of vsRNAs from *Cabbage leaf curl virus* (CaLCuV; a member of genus *Begomovirus* of the family *Geminiviridae*) was not affected, suggesting that RDR2 and RDR6 are not involved in production of dsRNA precursors of vsRNAs [7]. However, involvement of RDR1 in this process or possible redundancy in activities of distinct RDRs were not investigated so far.

Geminiviruses encapsidate circular single-stranded (ss)DNA of ca. 2.5-to-2.7 kb in geminate virions and accumulate in the nucleus as multiple circular dsDNA minichromosomes. The minichromosomes are both the intermediates of rolling circle replication and the templates for Pol II-mediated bidirectional transcription [32]. Like many members of the genus *Begomovirus*, CaLCuV has a bipartite genome comprising 2.6 kb DNA-A and 2.5 kb DNA-B [33]. The DNA-A encodes proteins involved in replication (AC1 and AC3), transcription (AC2) and encapsidation (AV1), while the DNA-B encodes BC1 and BV1 proteins with movement functions. A large intergenic region on DNA-A and DNA-B contains a 192 bp common region of nearly identical sequence with the origin of replication and bidirectional promoter elements. By analogy with other begomoviruses [34], the bidirectional promoter is expected to drive Pol II transcription of the leftward (*AC1/AC4/AC2/AC3* and *BC1*) and rightward (*AV1* and *BV1*) genes. In addition, a monodirectional promoter is expected to drive Pol II transcription of a short *AC2/AC3* transcript, which is co-terminal with the long *AC1/AC4/AC2/AC3* transcript. On both DNAs, the leftward and rightward transcription is terminated by poly(A) signals located in a close vicinity on the virion (sense) and complementary (antisense) strands, respectively. In CaLCuV DNA-A, this juxtaposition of the poly(A) signals creates a ca. 25-nt overlap of the sense and antisense transcripts. Such overlap was proposed to form a dsRNA precursor of primary vsRNAs [35], which may initiate RDR-dependent production of vsRNAs from other regions of the viral transcripts.

Such phenomenon of transitivity has been described for posttranscriptional and transcriptional silencing of a transgene targeted by vsRNAs (virus-induced gene silencing; VIGS) or by primary siRNAs derived from an inverted-repeat transgene. In these cases, RDR6- or RDR2-dependent production of secondary siRNAs outside of the target region was detected, respectively [36], [37]. Notably, posttranscriptional silencing of endogenous plant genes by virus- or transgene-derived primary siRNAs was not associated with secondary siRNA production [36], [38], [39], suggesting that endogenous mRNAs are not good templates for RDRs.

In this study, we used Illumina deep sequencing of short RNAs, combined with blot hybridization and genetic analysis, to investigate the biogenesis of primary and secondary siRNAs. To this end, *Arabidopsis* wild-type, *RDR*-mutant and transgenic plants were infected with CaLCuV or its derivatives carrying fragments of an endogenous gene or a transgene. We found that, like most endogenous plant mRNAs, viral mRNAs are not prone to transitivity: the majority of vsRNAs are RDR1-, RDR2- and RDR6-independent primary siRNAs. By contrast, a transgene mRNA targeted by primary vsRNAs is subject to RDR6-dependent production of secondary siRNAs. We also found that silencing of the transgene driven by a CaMV 35S promoter can be triggered by primary vsRNAs targeting an enhancer (but not core promoter) region and this, presumably transcriptional, silencing was not associated with accumulation of secondary siRNAs.

## Results/Discussion

### 21, 22 and 24 nt vsRNAs accumulate at high levels in CaLCuV-infected *Arabidopsis*

To analyze begomovirus interactions with the host small RNA (sRNA)-generating silencing pathways, we deep-sequenced sRNA populations from mock-inoculated and CaLCuV-infected *A. thaliana* wild-type (Col-0) plants and CaLCuV-infected *rdr1 rdr2 rdr6* triple null mutant plants (*rdr1/2/6* in Col-0 background; [8]). The protocol was designed to sequence short RNAs with 5′-phosphate and 3′-hydroxyl groups, which include DCL products. Samples of total RNA extracted from pools of three plants were processed in parallel and the resulting cDNA libraries sequenced in one channel of an Illumina Genome Analyzer, thus allowing quantitative comparison of changes in the profile of host sRNAs upon virus infection and the profile of vsRNAs in wild-type versus mutant plants.

A total number of reads in the high-coverage libraries was ranging from 9.3 to 10.4 million, of which 7.3 million (‘Col-0 mock’), 5.3 million (‘Col-0 CaLCuV’) and 5.0 million (‘*rdr1/2/6* CaLCuV’) of 20–25 nt reads mapped to the *Arabidopsis thaliana* Col-0 or CaLCuV genomes with zero mismatches (Table S1A). Two additional low-coverage libraries with 0.45 million (‘Col-0 mock*’) and 0.43 million (‘Col-0 CaLCuV*’) of 20–25 nt reads with zero mismatches (Table S1A) were obtained in an independent experiment.

**Figure 1:**
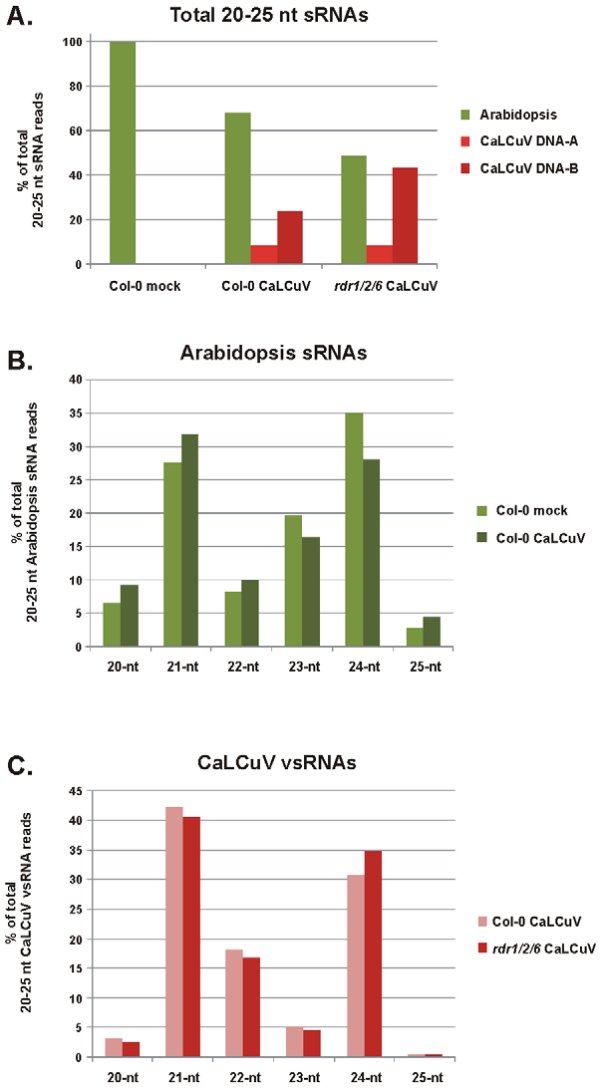
Illumina deep-sequencing of sRNAs from mock-inoculated and CaLCuV-infected *Arabidopsis* wild-type (Col-0) and *rdr1/2/6* triple mutant plants. The graphs show the percentages of *Arabidopsis* and vsRNAs in the pool of 20–25 nt reads mapped to the *Arabidopsis* and CaLCuV DNA-A and DNA-B genomes with zero mismatches (**A**), of each size-class of 20–25 nt host sRNA reads mapped to the *Arabidopsis* genome with zero mismatches (**B**), and of each size-class of 20–25 nt vsRNA reads mapped to the CaLCuV DNA-A and DNA-B with zero mismatches (**C**).

In mock-inoculated plants, most of the 20–25 nt sRNAs mapped to the *A. thaliana* genome (Figure 1A; Table S1A). The 24-nt and 21-nt classes were predominant (35% and 28%, respectively), whereas other size-classes were less abundant (23-nt – 19%; 22-nt – 8%; 20-nt – 7%; 25-nt – 3%) (Figure 1B). This is consistent with the previous studies showing that 24-nt hcsiRNAs and 21-nt miRNAs are the most abundant sRNA classes in *A. thaliana* [40], [41]. Upon CaLCuV infection, the host sRNA profile was slightly altered in that the 21-nt class became the largest (32%) and the 24-nt class the second largest (28%) (Figure 1B; Table S1A). A similar shift in the host sRNA profile was also detected in the low coverage experiment (Table S1A). By contrast, *A. thaliana* infection with the pararetrovirus CaMV results in overaccumulation of 24-nt host sRNAs [8]. The biological significance of the opposite effects of geminivirus and pararetrovirus infections on host sRNAs remains to be investigated.

**Figure 2:**
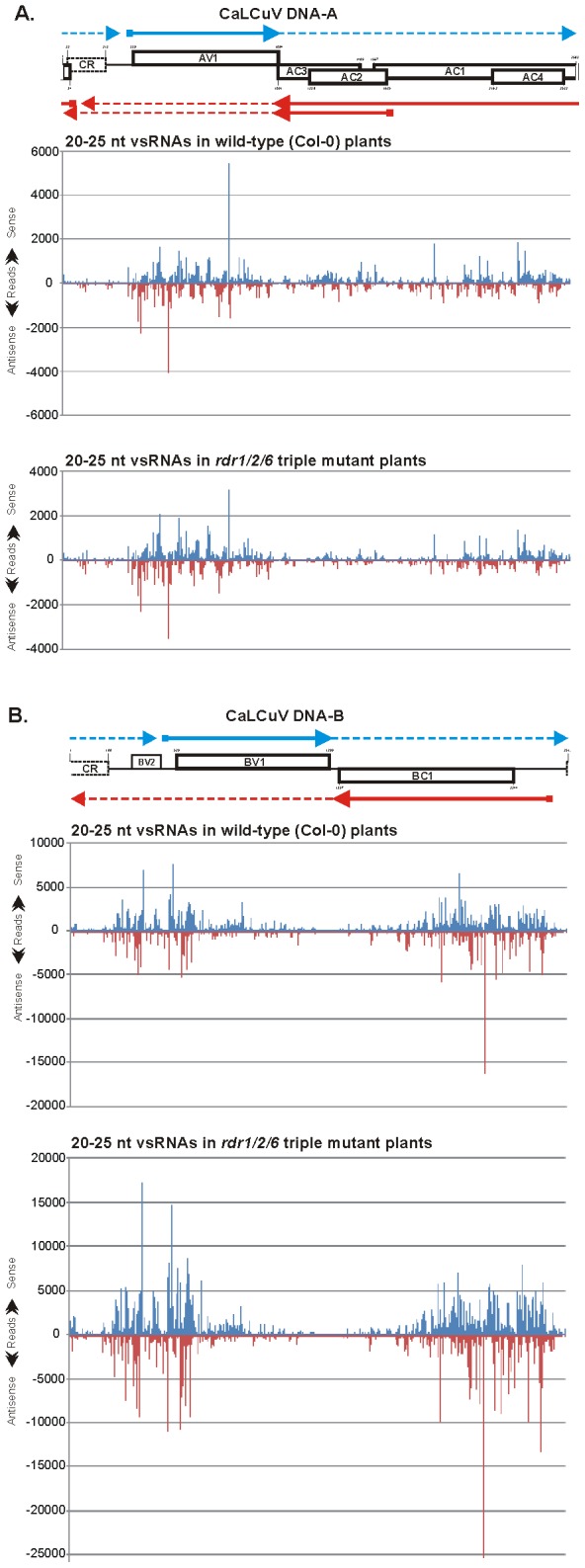
Maps of vsRNAs from CaLCuV-infected wild type (Col-0) and *rdr1/2/6* triple mutant plants at single-nucleotide resolution. The graphs plot the number of 20–25 nt vsRNA reads at each nucleotide position of the 2583 bp DNA-A (**A**) and the 2513 bp DNA-B (**B**); Bars above the axis represent sense reads starting at each respective position; those below represent antisense reads ending at the respective position (Tables S2 and S3). The genome organizations of DNA-A and DNA-B are shown schematically above the graphs, with leftward (AC1, AC4, AC2, AC3 and BC1) and rightward (AV1 and BV1) ORFs and common region (CR) indicated. The predicted rightward and rightward mRNAs are shown as respectively blue and red solid lines with arrowheads. Potential readthrough transcripts are shown as dotted thin lines.

In CaLCuV-infected Col-0 plants, a large fraction of 20–25 nt reads mapped to the virus genome with zero mismatches (ca. 32% and 62% in the high- and low-coverage libraries, respectively; Figure 1A and Table S1A). Notably, the viral DNA-B was the major source of vsRNAs (70% and 85% of 20–25 nt viral reads, respectively; Table S1A). On both DNA-A and DNA-B, vsRNA reads were almost equally distributed between the virion and complementary strands (Table S1A; Figures 2 and S1). Similar to the host sRNAs in infected plants, 21-nt and 24-nt vsRNAs represent the first (42%) and the second (31%) largest fractions of 20–25 nt viral reads, respectively. But unlike the host sRNAs, 22-nt viral reads represent the third largest fraction (18%), while 20-nt, 23-nt and 25-nt classes are significantly underrepresented (Figure 1C). This size-class profile of CaLCuV vsRNAs agrees with our blot hybridization analysis using short probes and confirms the involvement of distinct DCLs in vsRNA biogenesis (Figure S2; [7]).

Interestingly, the host sRNAs of 21-nt and 24-nt classes exhibit a strong bias to 5′-terminal uridine (5′U; 69%) and 5′-terminal adenosine (5′A; 52%), respectively (Table S1A), owing to the preferential association of miRNAs with AGO1 and hcsiRNAs with AGO4 [17], [42]–[44]. By contrast, vsRNAs of 21-nt and 24-nt classes are less strongly enriched in 5′U (46%) and 5′A (32%), respectively, and the second most dominant nucleotide is 5′A for 21-nt class (25%) and 5′U for 24-nt class (32%) (Table S1A). Both the diversity in nucleotide composition and size of CaLCuV vsRNAs and the lack of any strong 5′-nucleotide bias imply the involvement of multiple AGOs in sorting vsRNAs.

### vsRNA species densely tile along the entire circular viral DNAs and accumulate at high levels in several large hotspot regions

Inspection of single-nucleotide resolution maps of 20–25 nt vsRNAs revealed that unique vsRNA species of each major class (21-nt, 22-nt and 24-nt) cover the entire genome of CaLCuV in both sense and antisense polarity as dense tiling arrays without gaps on the circular sequences of 2583 bp DNA-A and 2513 bp DNA-B (Tables S2 and S3). Hence, dsRNA precursors of vsRNAs of each class should cover the entire circular viral DNAs. However, the relative abundance of vsRNAs varies drastically: several large regions of DNA-A and DNA-B are densely covered in both polarities with vsRNA hotspots (defined here arbitrarily as short sequence segments spawning several vsRNA species with more than 300 reads each) (Figure 2 and Figure S1). This implies the existence of several overlapping dsRNA precursors that accumulate at high and low levels. Interestingly, vsRNA hotspots on both virion and complementary strands are interrupted with short sequences that spawn vsRNAs of lower abundance (Figure 2 and Figure S1; Table S2 and Table S3). This implies differential stability of vsRNA duplexes processed consequently from ends of long dsRNA precursors or, alternatively, preferential internal excisions of vsRNA duplexes from certain regions of a long dsRNA. We also found that most vsRNA hotspots contain all the three major size-classes of vsRNAs (Figure S1; Table S2 and Table S3), indicating that same dsRNA precursors are processed by different DCLs. This conclusion is consistent with our genetic analysis coupled with blot-hybridization of DNA virus-derived sRNAs [6], [7] (Figure S2) and sRNA deep-sequencing studies of other viruses [8], [45]–[48].

In DNA-A, the most abundant vsRNAs of both sense and antisense polarities, which include those with more than 1000 reads, originate from the *AV1* ORF (Figure 2A and Figure S1A). The left border of this vsRNA hotspot region is at position 331 (Table S2), where the transcription start site can be predicted, i.e. at an optimal distance downstream of the TATA box (TATATAA at positions 228–305) and 9 nts upstream of the *AV1* start codon (339–341). The right border of this vsRNA hotspot is at around position 1060 (Table S2), i.e. just upstream of the *AV1* stop codon (1092–1094). After a short gap of 55 bp (1061–1116) lacking highly abundant vsRNAs, a large region spanning all the leftward ORFs is also covered with vsRNA hotspots, albeit at lower density than in the *AV1* region. In this region, the most abundant vsRNAs originate from the large portion of the *AC1* ORF including the nested AC4 ORF and less abundant vsRNAs from the *AC2* ORF (Figure 2A; Table S2). Notably, the 25 nt region (1089–1113), in which the rightward (*AV1*) and the leftward (*AC1/AC4/AC2/AC3* and *AC2/AC3*) viral mRNAs are expected to overlap and potentially form a dsRNA substrate for DCL, is not a vsRNA hotspot. Likewise, the 240 bp intergenic region between the predicted leftward and rightward transcription start sites (at positions 93 and 331, respectively), which contains the bidirectional promoter elements and overlaps the common region (22–213), is also devoid of vsRNA hotspots: it has only two islands covered with vsRNAs of 100–250 reads. Furthermore, the promoter region in front of the predicted transcription start site of *AC2/AC3* mRNA (position 1651, downstream of TATATAA at 1683–1677) does not contain any prominent vsRNA hotspots (Figure 2A and Figure S1A; Table S2). Taken together, the promoter and terminator regions of CaLCuV DNA-A are devoid of highly abundant vsRNAs. Thus, the virus may have evolved a mechanism to evade transcriptional silencing which could potentially be directed by vsRNAs.

In DNA-B, two large regions are covered with extreme hotspots containing multiple vsRNA species with more than 1000 reads on both sense and antisense strands. The first is located downstream of the common region and it spans a large portion of the *BV1* ORF. The second is located upstream of the common region and it spans a large portion of the *BC1* ORF (Figure 2B and Figure S1B; Table S3). Like in DNA-A, the terminator region of rightward (*BV1*) and leftward (*BC1*) genes is devoid of vsRNA hotspots. Note that the DNA-B poly(A) signals AATAAA are located at positions 1305–1310 and 1356–1361 of the virion and complementary stands, respectively, and therefore the *BV1* and *BC1* mRNAs are not expected to overlap. A predicted BC1 promoter region with the TATA-box at positions 2471–2463 (TATATAA) is devoid of vsRNA hotspots and the border of the vsRNA hotspot region corresponds to the predicted transcription start site at 2439. Thus, *BC1* mRNA can form one of the strands of a vsRNA precursor. In contrast, a predicted *BV1* promoter region with the TATA-box at position 442–447 (TATATAA) is covered with vsRNA hotspots on both strands. This suggests that the region upstream of the *BV1* ORF might be actively transcribed. Interestingly, it contains an ORF at positions 319 to 471 (Figure 2B). Such active transcription could in turn lead to production of abundant vsRNAs that can potentially direct transcriptional silencing of the *BV1* promoter. This may represent either a host antiviral defense or a viral strategy of gene regulation.

Based on close inspection of cold versus hot spots of viral siRNAs, AU-rich sequences can generally be considered as a poor source of siRNAs, possibly owing to relatively low stability of AU-rich siRNA duplexes processed by DCLs from long dsRNA precursors. Other features of RNA primary or secondary structure which might potentially influence siRNA biogenesis or stability remain to be further investigated.

### vsRNA biogenesis is not affected drastically in plants lacking RDR1, RDR2 and RDR6

The *Arabidopsis* sRNA profile is drastically altered in *rdr1/2/6* triple mutant compared to wild-type plants: 24-nt and 23-nt classes are selectively and strongly reduced, mainly owing to the loss of RDR2-dependent hcsiRNAs [40]. Thus, 21-nt class becomes the most predominant, followed by 20-nt and 22-nt classes (Table S1A): these three classes are mainly populated with RDR-independent miRNAs, whereas RDR6-dependent tasiRNAs and secondary siRNAs are much less abundant [41]. By contrast, the CaLCuV vsRNA profile was only slightly altered in *rdr1/2/6* compared to wild-type (Figure 1C).

The overall accumulation level of 20–25 nt vsRNAs was higher in *rdr1/2/6* than wild-type plants. If normalized by the levels of 21-nt host sRNAs (1.22 million in ‘Col-0 CaLCuV’ versus 1.21 million in ‘*rdr1/2/6* CaLCuV’), this ca. 1.5-fold increase is mainly owing to higher accumulation of DNA-B vsRNAs of all the major classes (Table S1A; Figure 1A).

The single-nucleotide resolution maps of vsRNAs from Col-0 and *rdr1/2/6* are remarkably similar. The vsRNA hotspots occur in the same regions and the relative abundance of vsRNA species is very similar within most hotspots (Figure 2 and Figure S1; Table S2 and Table S3). For DNA-A, the levels of 20–25 nt vsRNAs derived from the *AC2* hotspot region are relatively lower in *rdr1/2/6* than in Col-0, whereas those derived from the *AV1* region are generally similar in *rdr1/2/6* and Col-0 (Figure 2A), with an exception of 24-nt vsRNAs that accumulate at relatively higher levels in *rdr1/2/6* (Figure S1A; Table S1A). For DNA-B, the levels of 20–25 nt vsRNAs in most hotspots are 1.5- to 2.5-fold higher in *rdr1/2/6* than in Col-0, with an exception of the middle part and the 3′ part of *BV1* ORF, in which vsRNA levels are generally similar in *rdr1/2/6* and Col-0 or, at some locations in the 3′ part, lower in *rdr1/2/6* (Figure 2B). No drastic difference in the relative abundance of vsRNA size-classes along the DNA-B sequence was observed (Figure S2B; Table S3).

Analysis of 5′-terminal nucleotides of vsRNAs revealed no substantial difference between Col-0 and *rdr1/2/6* (Table S1A), further supporting that vsRNA biogenesis is not drastically affected by null mutations in *RDR1*, *RDR2* and *RDR6*.

**Figure 3:**
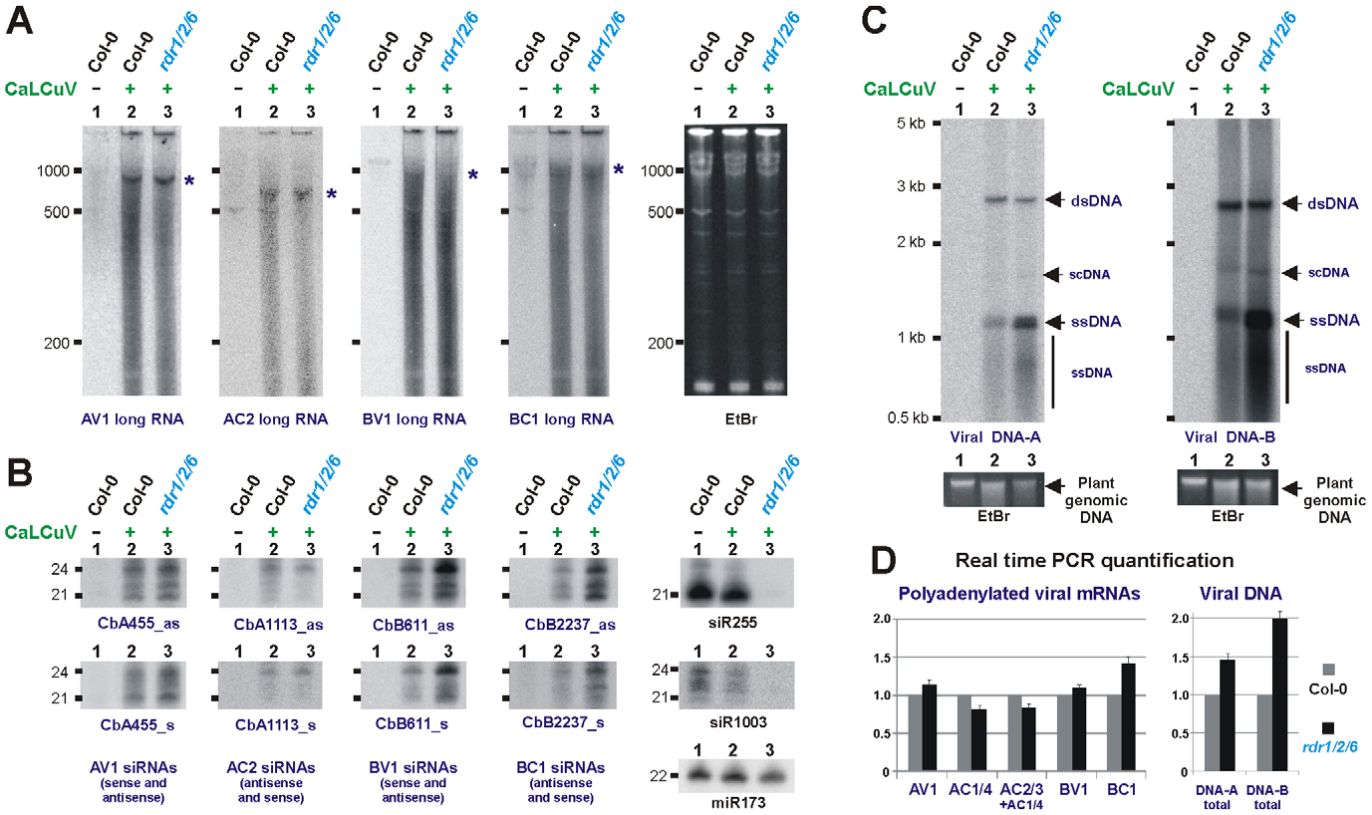
Accumulation of long viral nucleic acids and vsRNAs in wild type versus *rdr1/2/6* triple mutant plants. Total RNA and total DNA from CaLCuV-infected *Arabidopsis* wt (Col-0) and *rdr1/2/6* plants was analyzed by RNA blot hybridization using 5% (**A**) and 15% (**B**) PAGE and by Southern blot hybridization (**C**). The RNA blot membranes were successively hybridized with mixtures of DNA oligonucleotide probes complementary to respective viral mRNAs (for sequences, see Protocol S1) and, in the case of sRNA analysis, single DNA oligonucleotide probes specific for vsRNA of sense or antisense polarity and the endogenous *Arabidopsis* miRNA (22 nt miR173), tasiRNA (21 nt siR255) and hcsiRNA (24 nt siR1003). The Southern blot membranes were hybridized with long dsDNA probes specific for DNA-A or DNA-B. Positions of co-migrating forms of viral DNA including open-circular double-stranded (dsDNA), supercoiled (scDNA) and single-stranded (ssDNA) are indicated by arrows; the smear of shorter (than monomeric) ssDNA is also indicated. EtBr staining of total RNA (**A**) or plant genomic DNA (**C**) is shown as loading control. The size markers are indicated on each scan. Positions of viral mRNAs are indicated by asterisks. (**D**) Real time qPCR measurement of relative accumulation of viral polyadenylated mRNAs (left) and total viral DNAs A and B (right) in wild type versus *rdr/1/2/6* mutant plants. For each mRNA and each DNA, the accumulation level in the wild type sample is set to 1.

The above-described deep sequencing findings for vsRNA size-classes, relative abundance and distribution along the viral genome and RDR1/2/6-independence of vsRNA biogenesis were confirmed by blot hybridization analysis of sRNAs from CaLCuV-infected wild-type and *rdr1/2/6* mutant plants using several short probes specific to DNA-A or DNA-B (Figure S2 and Figure 3B). In addition, analysis of CaLCuV-infected *dcl1 dcl2 dcl3 dcl4* quadruple mutant plants (*dcl1/2/3/4*) confirmed our previous findings that the majority of vsRNAs are generated by four DCLs [7]. We further established that a mutant DCL1 protein produced from the *dcl1-9*/*caf1* allele in *dcl1/2/3/4* plants [8] appears to be capable of generating 21-nt vsRNA from dsRNA precursors derived from vsRNA hotspot regions of DNA-B (Figure S2). Likewise, a major fraction of 21-nt vsRNAs derived from the leader region of CaMV, which is an extreme hotspot of 21-24 nt vsRNA production, requires DCL1 for their biogenesis and residual accumulation of 21-nt vsRNAs was observed in *dcl1/2/3/4* [8].

Taken together, our findings indicate that CaLCuV vsRNA biogenesis does not require RDR1, RDR2, or RDR6. However, there appears to be a quantitative difference in relative abundance of dsRNA precursors derived from the vsRNA hotspot regions of DNA-A and DNA-B in wild-type versus *rdr1/2/6* plants.

### Accumulation of viral long nucleic acids in wild type versus *rdr1/2/6* plants

To test if the observed differences in relative abundance of vsRNAs correlate with relative levels of viral transcripts and/or viral DNA, we measured the accumulation of viral long nucleic acids in wild-type and *rdr1/2/6* plants by RNA and DNA blot hybridization as well as real time PCR (Figure 3). The results of total RNA (Figure 3A) and polyadenylated mRNA (Figure 3D) analyses revealed that the relative accumulation of viral transcripts positively correlates the relative abundance of vsRNAs in the major hot spot regions. Indeed, AV1 mRNA, the most readily detectable viral transcript, accumulated at slightly higher levels in *rdr1/2/6* than wild type plants, whereas accumulation of the less abundant AC2/AC3 mRNA was slightly reduced in *rdr1/2/6*. This resembles the profile of DNA-A derived vsRNAs and its alteration in *rdr1/2/6*. Furthermore, accumulation of BC1 and BV1 polyadenylated mRNAs was increased ca. 1.2- and 1.4-fold, respectively, in *rdr1/2/6* compared to wild type plants, which correlates with slightly increased accumulation of DNA-B derived vsRNAs in *rdr1/2/6*. Notably, in addition to viral mRNAs, shorter viral transcripts also accumulate at high levels and appear as a smear on the total RNA blot (Figure 3A). These aberrant RNAs may represent degradation products of viral mRNAs or prematurely terminated viral transcripts. In the case of DNA-B, the aberrant RNAs appear to be much more abundant than BV1 and BC1 mRNAs, since the latter are barely detectable (Figure 3A). This correlates with much higher accumulation of vsRNAs from DNA-B than DNA-A (Figure 1A). The higher abundance of aberrant RNAs transcribed from DNA-B can be explained by higher accumulation of total DNA-B compared to total DNA-A as estimated by Southern (Figure 3C).

Real time PCR analysis (Figure 3D) revealed that total viral DNA accumulates at higher levels in *rdr1/2/6* compared to wild type plants (ca. 1.4- and 2-fold increase for DNA-A and DNA-B, respectively). However, Southern blot hybridization analysis (Figure 3C) showed that this increase is mainly owing to increased accumulation of viral single-stranded DNA (ssDNA). By contrast, the levels of viral dsDNA, which serves as a template for both transcription and replication, are similar in wild type and *rdr1/2/6* plants. Thus, rolling circle and/or recombination-dependent replication mechanisms [32] produce increased levels of viral ssDNA (but not dsDNA) in the absence of RDR1, RDR2 and RDR6. This finding implicates an RDR activity in the regulation of geminiviral DNA replication. Interestingly, homologous recombination-dependent, double-stranded DNA brake (DSB) repair in *Arabidopsis* involves DSB-induced small RNAs (diRNAs) [49]. RDR2 and RDR6 play redundant roles in the biogenesis of diRNAs, implicating RDR activity in DSB repair.

### Silencing of a host gene directed by CaLCuV-derived primary siRNAs is not associated with production of secondary siRNAs

Our above-described results suggested that CaLCuV vsRNAs are primary siRNAs (i.e. RDR-independent) and that secondary siRNAs (i.e. RDR-dependent) may comprise only a small fraction of vsRNAs (if any). To investigate if primary vsRNAs are capable of triggering production of secondary siRNAs in CaLCuV-infected plants, we used a virus-induced gene silencing (VIGS) vector based on the CaLCuV DNA-A derivative lacking most of the *AV1* ORF sequence (positions 350–1032) [50].

When a 354 bp fragment of the *A. thaliana Chlorata I* (*ChlI/CH42*; At4g18480) gene ORF is inserted in place of the AV1 ORF, the resulting recombinant virus CaLCuV::Chl knocks down *ChlI* mRNA levels in all tissues of CaLCuV::Chl-infected *A. thaliana* plants [7] and causes whitening of newly growing tissues due to the loss of chlorophyll (“*chlorata”* phenotype; [50]). The recombinant virus spawns abundant 21, 22, and 24 nt siRNAs from the *ChlI* insert, whose biogenesis does not require RDR6 or RDR2. However, an extensive chlorata phenotype is nearly abolished in *rdr6* and *dcl4* null mutant plants [7], suggesting that RDR6-/DCL4-dependent secondary siRNAs might be involved in total silencing the *ChlI* gene. To test this hypothesis we deep-sequenced sRNAs from CaLCuV::Chl-infected Col-0 plants exhibiting an extensive chlorata phenotype.

Of 2.28 million total 20–25 nt reads, 1.58 million mapped to the *A. thaliana* genome and 0.61 million to CaLCuV::Chl genome (A+B) with zero mismatches. Of the latter reads, 0.45 million originate from the circular CaLCuV::Chl DNA and 0.16 million from DNA-B (Table S1B). This is in contrast to our above observation for wild-type CaLCuV which spawns more abundant vsRNAs from DNA-B.

**Figure 4:**
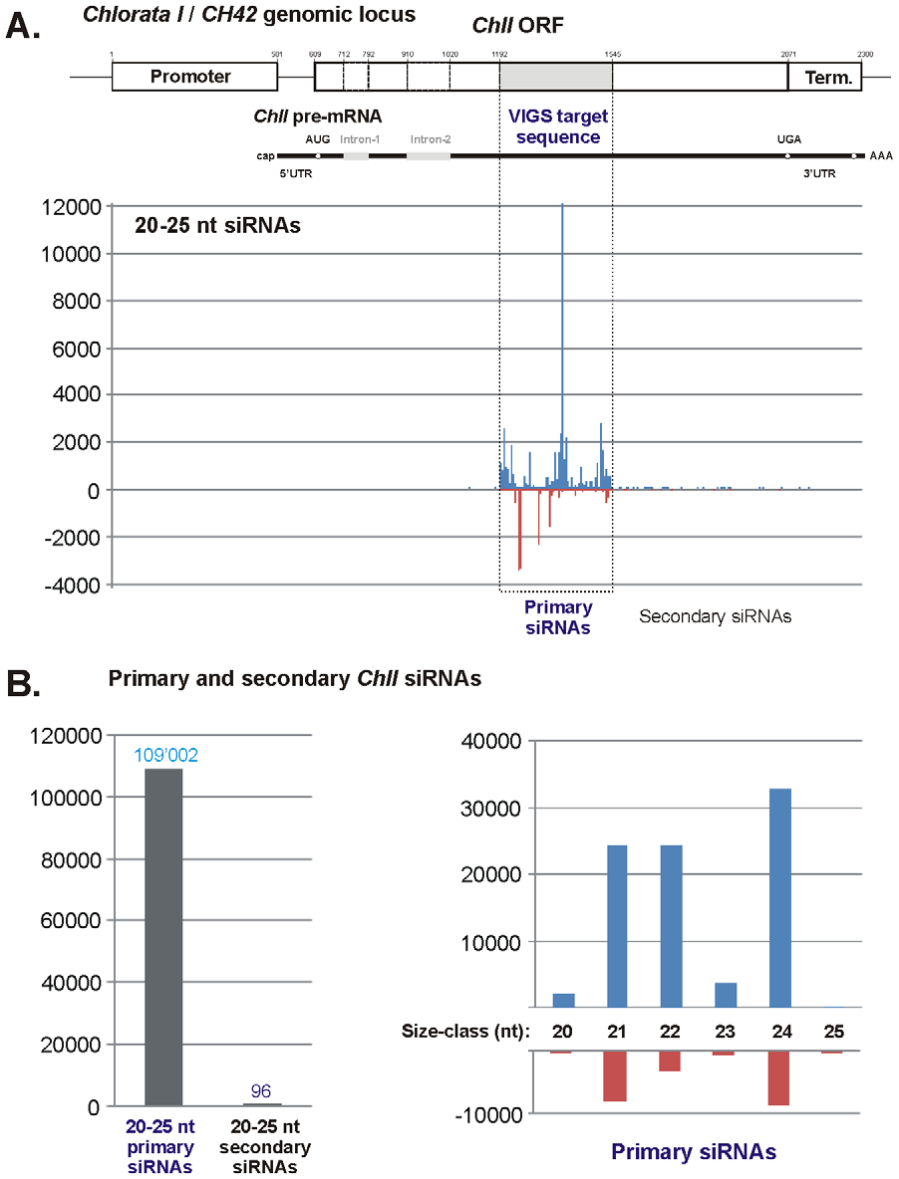
Primary and secondary siRNAs in CaLCuV::Chl virus-infected wild type (Col-0) plants. (**A**) The 2300 bp region of the *Arabidopsis* genome, which contains *Chlorata I/CH42* gene (*ChlI*), is shown schematically with positions of *ChlI* promoter, pre-mRNA with two introns, and terminator sequences indicated; numbering starts 500 nucleotides upstream of the transcription start site. The VIGS target sequence (inserted in CaLCuV::Chl virus) is highlighted with grey. The graph plots the number of 20–25 nt siRNA reads at each nucleotide position of the *ChlI* gene; Bars above the axis represent sense reads starting at each respective position; those below represent antisense reads ending at the respective position (Table S4). (**B**) The left bar graph shows the total numbers of 20–25 nt primary (CaLCuV::Chl-derived) and secondary siRNAs derived from *ChlI* sequences outside of the VIGS target region, while the right bar graph shows the number of primary siRNAs for each size class and polarity.

Inspection of the single-nucleotide resolution map of 20–25 nt sRNAs perfectly matching to a 3298 bp region of the *A. thaliana* genome, which contains the *ChlI* gene, revealed that of the 109′098 redundant reads, 109′002 originate from the 354 bp segment (positions 1192–1545) that corresponds exactly to the *ChlI* segment inserted in CaLCuV::Chl. The remaining sRNAs (91 reads) originate mostly from the *ChlI* sequence downstream of this segment (Figure 4; Table S1B and Table S4). We conclude that accumulation of secondary siRNAs outside of the vsRNA target region is negligible compared to primary siRNAs. This is consistent with the previous studies that detected no transitivity when endogenous plant genes were knocked down by RNA virus- or transgene-induced silencing [36], [38], [39].

Within the *ChlI* target region the sRNA profile resembles the global profile of CaLCuV vsRNAs in that the three size-classes are predominant (21-nt – 30%; 22-nt – 25%; 24-nt – 38%). However, the distribution of sRNAs is unequal between the strands: 80% of 20–25 nt reads map to the coding strand, and 21-nt and 22-nt classes derived from the coding strand are equally abundant (28% each). This strong bias is due to a bigger number of sRNA hotspots and higher accumulation levels of sRNA species within the hotspots on the coding strand (Figure 4; Table S4). The significance of this bias for *ChlI* silencing remains to be investigated.

In *A. thaliana*, the *ChlI* gene has a close homolog *ChlI-2* (At5g45930), silencing of which is likely required for the chlorata phenotype. To address if potential silencing of *ChlI-2* is associated with secondary siRNA production we created a map of *ChlI-2* sRNAs (Figure S3A). Of 3′093 reads of 20–25 nt sRNAs matching the *ChlI-2* genomic locus with zero mismatches in CaLCuV::Chl-infected plants, 2′987 reads map within the 354 bp VIGS-target sequence and only 104 (ca. 3%) map downstream of the target. Moreover, within the target sequence almost all the reads (2′977) match two sequence stretches of >20 nts in length which are identical in *ChlI* and *ChlI-2* (Figure S3A; Table S4). Thus, similar to *ChlI*, only small amounts of secondary siRNAs are generated on *ChlI-2* target gene. Presently, we cannot exclude that these small amounts of secondary siRNAs are required for total chlorata silencing. As we hypothesized earlier [7], total *Chl* silencing is likely established in newly emerging leaves by mobile RDR6- and DCL4-dependent *Chl* siRNAs. Recent studies indicate that 21–24 nt siRNAs act as mobile silencing signals and can direct mRNA cleavage and DNA methylation in recipient cells, even though they accumulate in recipient tissues at much lower levels than in source tissues [51], [52].

Notably, vsRNAs targeting *ChlI-2* mRNA at two potentially cleavable sites separated by ca. 100 nts do not trigger any robust secondary siRNA production from the intervening region. This indicates that a two-hit model for the RDR6-dependent biogenesis of tasiRNAs and other secondary siRNAs [14], [19], [53] does not apply for *ChlI-2* and *ChlI*.

Like in the wild-type DNA-A, vsRNAs cover the entire circular CaLCuV::Chl DNA in both orientations without gaps (Table S4). However, vsRNA hotspots are more evenly distributed along the CaLCuV::Chl sequence compared to the wild-type DNA-A: in fact, new hotspots appear in the intergenic region between the transcription start sites as well as in the terminator region (Figure S3; Table S4). This finding was confirmed by blot hybridization (Figure S2, compare CaLCuV wt and CaLCuV::Chl). Furthermore, genetic analysis revealed that production of vsRNAs from any region of CaLCuV::Chl including the *ChlI* insert does not require RDR6 or RDR2, since vsRNAs of all classes accumulated at similar levels in wild type and *rdr2 rdr6* double mutant plants (*rdr2/6*; Figure S2). The latter finding indicates that RDR6-dependent secondary siRNA production does not occur within the VIGS target region and that potential cleavage of endogenous (*ChlI* or *ChlI-2*) and CaLCuV mRNAs at two sites is not sufficient to attract RDR6 activity.

Taken together, our findings for both wild-type and CaLCuV::Chl viruses suggest that dsRNA precursors of vsRNAs originate from the entire circular viral DNAs including “non-transcribed” intergenic regions. Therefore, these precursors might be produced by Pol II-mediated readthrough transcription far beyond the poly(A) signals, thus encircling the viral DNA in sense and antisense orientation. It can be further suggested that such readthrough transcription is more efficient on CaLCuV::Chl DNA-A than wild-type DNA-A, owing to the smaller size and the chimeric configuration of the rightward transcription unit carrying the *ChlI* segment. This would explain prominent hotspots in the promoter and terminator regions and also much higher production of vsRNAs from CaLCuV::Chl DNA-A than DNA-B, which is not the case for wild-type CaLCuV. Notably, CaLCuV::Chl is an attenuated virus which produces much less severe symptoms than wild type CaLCuV [49]. Whether vsRNA-directed silencing contributes to the attenuated symptom development of this recombinant virus remains to be investigated.

### Targeting a transgene transcribed region by CaLCuV-derived primary siRNAs triggers robust production of secondary siRNAs

**Figure 5:**
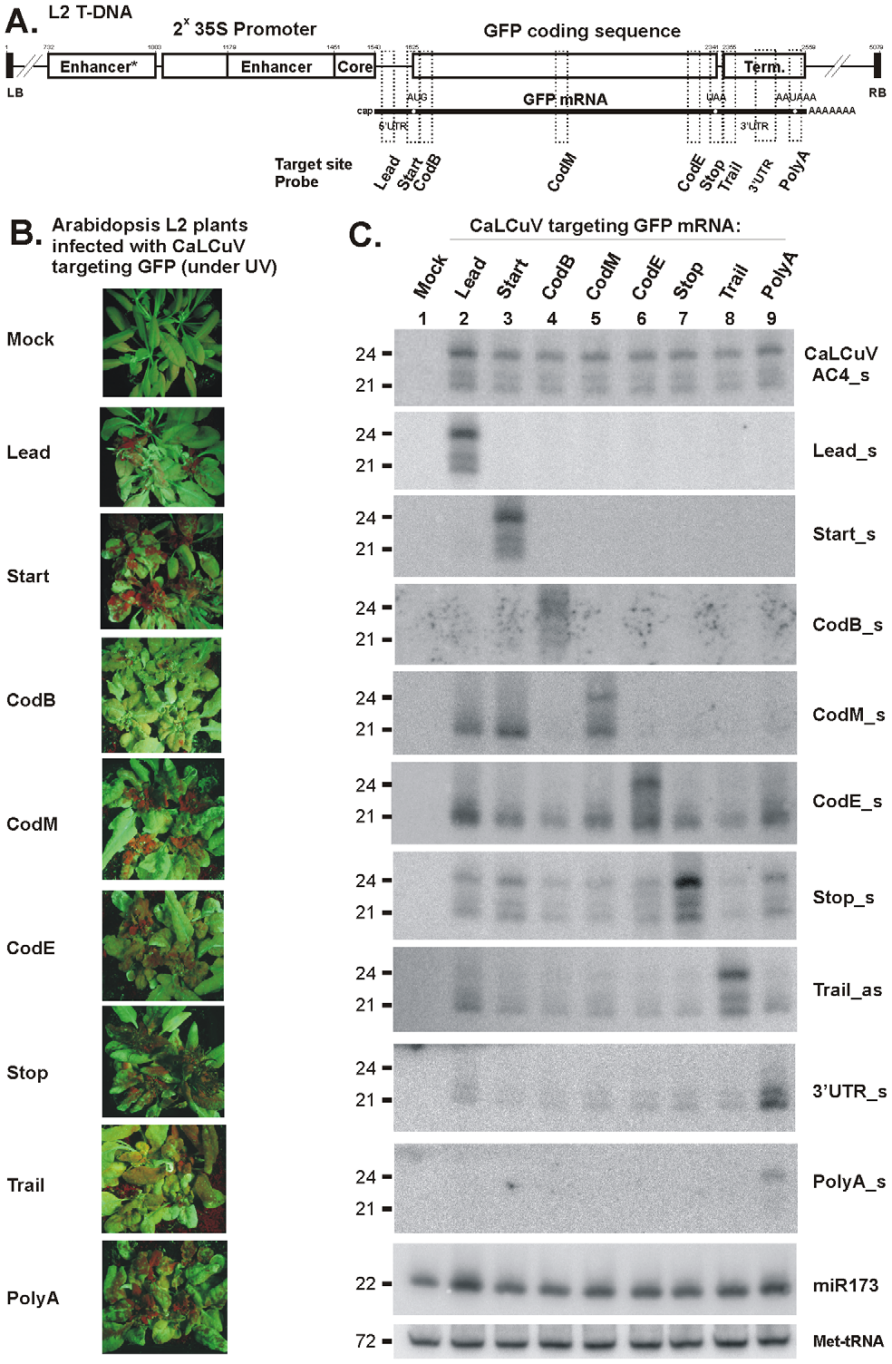
VIGS phenotypes and accumulation of primary and secondary siRNAs in L2 transgenic plants infected with CaLCuV::GFP viruses targeting the *GFP* transcribed region. (**A**) The L2 T-DNA region containing the 35S-GFP transgene is shown schematically. Positions of the duplicated CaMV 35S enhancer and core promoter elements, *GFP* mRNA elements including 5′UTR, translation start (AUG) and stop (UAA) codons and 3′UTR with poly(A) signal (AAUAAA), and 35S terminator sequences indicated. Numbering is from the T-DNA left border (LB). The VIGS target sequences, inserted in the CaLCuV::GFP viruses *Lead*, *CodB, CodM, CodE, Trail* and *polyA*, are indicated with dotted boxes; (**B**) Pictures under UV light of L2 transgenic plants infected with the above viruses; (**C**) Blot hybridization analysis of total RNA isolated from plants shown in Panel B. The blot was successively hybridized with short DNA probes specific for CaLCuV *AC4* gene (AC4_s) and 35S::GFP transgene sequences inserted in the CaLCuV::GFP viruses (*Lead, CodB, CodM, CodE, Trail* and *polyA*), the *GFP* mRNA 3′UTR non-target sequence (3′UTR) and *Arabidopsis* miR173 and Met-tRNA (the latter two serve as loading control).

The apparent paucity of secondary siRNAs derived from CaLCuV mRNAs or *ChlI* and *ChlI-2* mRNAs could be explained by two scenarios. In the first scenario, the products of potential vsRNA-directed cleavage of host and viral mRNAs are not optimal templates for RDR activity. In the second one, CaLCuV infection blocks RDR activity and thereby prevents RDR-dependent amplification of siRNAs. To distinguish between these scenarios, we used the CaLCuV VIGS vector for targeting a transgene in the *A. thaliana* line L2 expressing green fluorescence protein (GFP) under the control of the CaMV 35S promoter and terminator (35S::GFP; [54]; Figure 5). Like other transgenes, 35S promoter-driven *GFP* transgenes in *A. thaliana* and *N. benthamiana* are prone to transitivity in which secondary siRNAs are generated outside of the region targeted by primary sRNAs [36], [38], [55]. An aberrant nature of transgenic transcripts appears to attract RDR activity.

We inserted in the CaLCuV vector a full-length (FL), 771 bp *GFP* coding sequence (designated ‘*CodFL*’) or 30-bp sequences of the *GFP* transgene transcribed region. The latter is defined here as the *GFP* mRNA region from the transcription start site to the mRNA processing/poly(A) addition site. As depicted in Figure 5, the short inserts included the sequences from within the 5′-untranslated region (5′UTR) (designated ‘*Lead*’), the beginning, middle and end of the coding sequence (‘*CodB*’, ‘*CodM*’ and ‘*CodE*’), and the 3′UTR (‘*Trail*’ and ‘*PolyA*’) and the sequences surrounding the ATG start codon (‘*Start*’) or the TAA stop codon (‘*Stop*’). Inoculation of L2 plants with the resulting recombinant viruses by biolistic delivery of viral DNA led to development of local *GFP* silencing on inoculated leaves followed by systemic *GFP* silencing on newly-emerging infected tissues (both leaves and inflorescence; Figure S4B). *GFP* silencing in infected tissues, which was manifested under UV light as red fluorescence areas on otherwise green fluorescent tissues (Figure 5B and Figure S4B), well correlated with knockdown of *GFP* mRNA levels as measured by real time PCR (Figure S4D).

All the recombinant viruses carrying an insert from the *GFP* transcribed region induced systemic *GFP* silencing, although to various degrees (Figure 5B). Furthermore, in all these cases, *GFP* silencing correlated with accumulation of *GFP* siRNAs derived from both the short insert/target sequences and the *GFP* mRNA sequences outside of the target sequence (Figure 5C and Figure S4C). Notably, the 30 bp *GFP* insert/target sequences generally gave rise to abundant siRNAs of 21-nt, 22-nt and 24-nt classes, resembling those derived from the virus genome and therefore likely originating from the replicating virus carrying the insert rather than from the transgene. By contrast, secondary siRNAs derived from non-target sequences of the *GFP* transgene were generally represented by a dominant 21-nt class, although 22-nt and 24-nt classes were also detected (Figure 5C; also see below). Furthermore, targeting the *GFP* sequences upstream of the translation stop codon (*Lead, Start, CodB, CodM* and *CodE*) induced the production of abundant secondary siRNAs exclusively from sequences downstream of the target site, whereas targeting the 3′UTR sequences (*Stop, Trail* and *PolyA*) resulted in secondary siRNAs from the sequences upstream and downstream of the target site (Figure 5C). Such directionality in secondary siRNA biogenesis resembles that in RDR6-/DCL4-dependent biogenesis of tasiRNAs [17], [18]. Our findings further suggest that, following vsRNA-directed cleavage of *GFP* mRNA, the 5′-cleavage product might be protected by translating ribosomes from being converted to dsRNA precursor of secondary siRNAs. However, if it contains the translation stop codon, the ribosomes can terminate translation and be released. Thus, following vsRNA-directed cleavage downstream of the stop codon, both 5′ and 3′ cleavage products of *GFP* mRNA enter the secondary siRNA-generating pathway.

**Figure 6:**
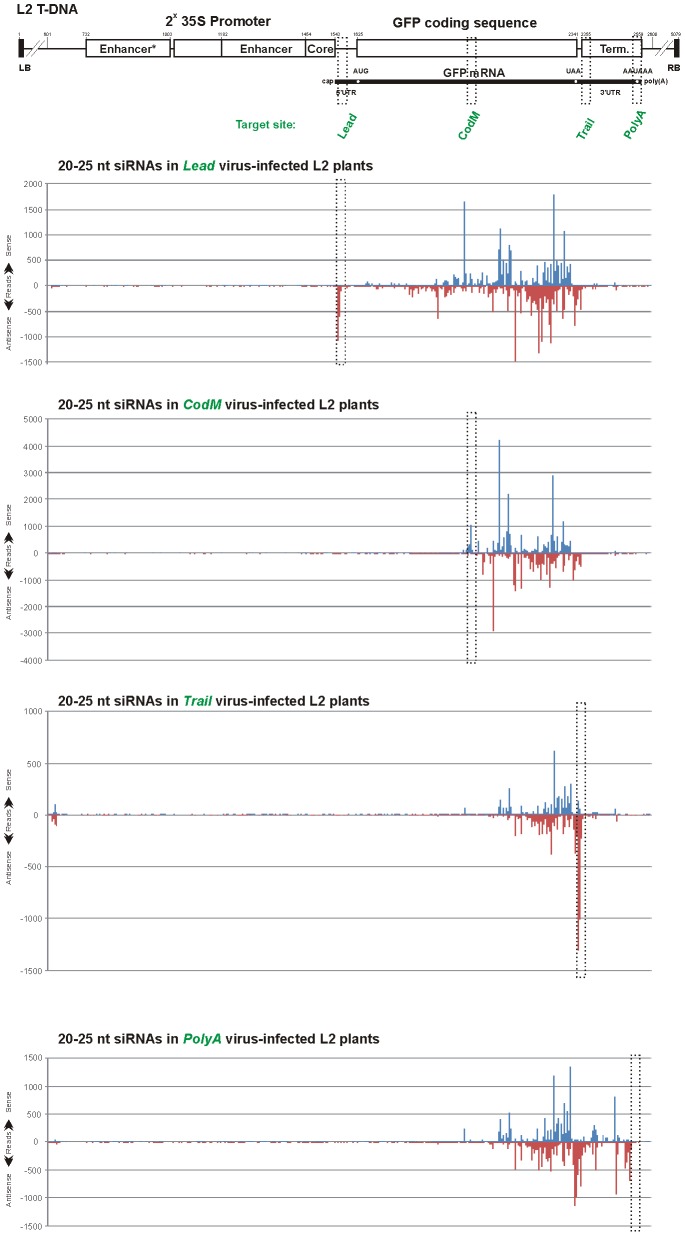
Maps of primary and secondary siRNAs accumulating in L2 transgenic plants infected with CaLCuV::GFP viruses that target the *GFP* transcribed region. The graphs plot the number of 20–25 nt vsRNA reads at each nucleotide position of the L2 T-DNA-based 35S::GFP transgene; Bars above the axis represent sense reads starting at each respective position; those below represent antisense reads ending at the respective position (Table S5). The 35S-GFP transgene is shown schematically above the graphs. Positions of the duplicated 35S enhancer and core promoter, *GFP* mRNA elements and 35S terminator are indicated. Numbering is from the T-DNA left border (LB). The VIGS target sequences inserted in the CaLCuV::GFP viruses Lead, CodM, Trail and polyA are indicated with dotted boxes.

The above findings based on blot hybridization analysis (Figure 5C) were fully validated by Illumina sequencing of sRNAs from L2 plants infected with *Lead, CodM, Trail* and *polyA* viruses (Figure 6 and Figure S5; Table S5 and Table S6). In addition, analysis of the deep sequencing data showed that vsRNAs targeting the 3′UTR induce production of much more abundant secondary siRNAs from the region upstream of the target site than from downstream sequences (Figure 6). Interestingly, secondary siRNA hotspots are non-randomly distributed along the *GFP* transcribed region: in all the four cases the siRNA hotspots occur in the region comprising the 3′ portion of the *GFP* ORF and the beginning of the 3′UTR. The size-class profile and relative abundance of siRNA species in this siRNA hotspot region are very similar. In the case of *Lead* and *polyA* viruses, additional siRNA hotspots occur in the middle of *GFP* ORF and the 3′UTR, respectively (Figure 6 and Figure S5). Interestingly, vsRNAs targeting the 5′UTR does not induce abundant secondary siRNA production from the region immediately downstream of the target site, which contains the 5′ portion of *GFP* ORF. This region also appears to be a poor source/target of primary vsRNAs (see *CodB* in Figure 5). Furthermore, robust production of secondary siRNAs does not appear to depend on the accumulation levels of any major size-class of primary vsRNAs of antisense polarity that have the potential to cleave *GFP* mRNA and initiate secondary siRNA biogenesis (Figure S5; Table S1, Table S5 and Table S6). We assume that, once initiated by primary vsRNAs, secondary siRNA biogenesis might be reinforced by feedback loops in which certain secondary siRNAs of antisense polarity target the *GFP* mRNA. Such feedback loops regulate tasiRNA production from *TAS1c* gene, in which certain tasiRNAs cleave its own precursor transcript to initiate RDR6-dependent production of additional dsRNAs [20], and potentially occur in transgene-induced silencing systems [56], [57].

### Targeting a transgene enhancer region by CaLCuV-derived primary siRNAs causes silencing without secondary siRNA production

**Figure 7:**
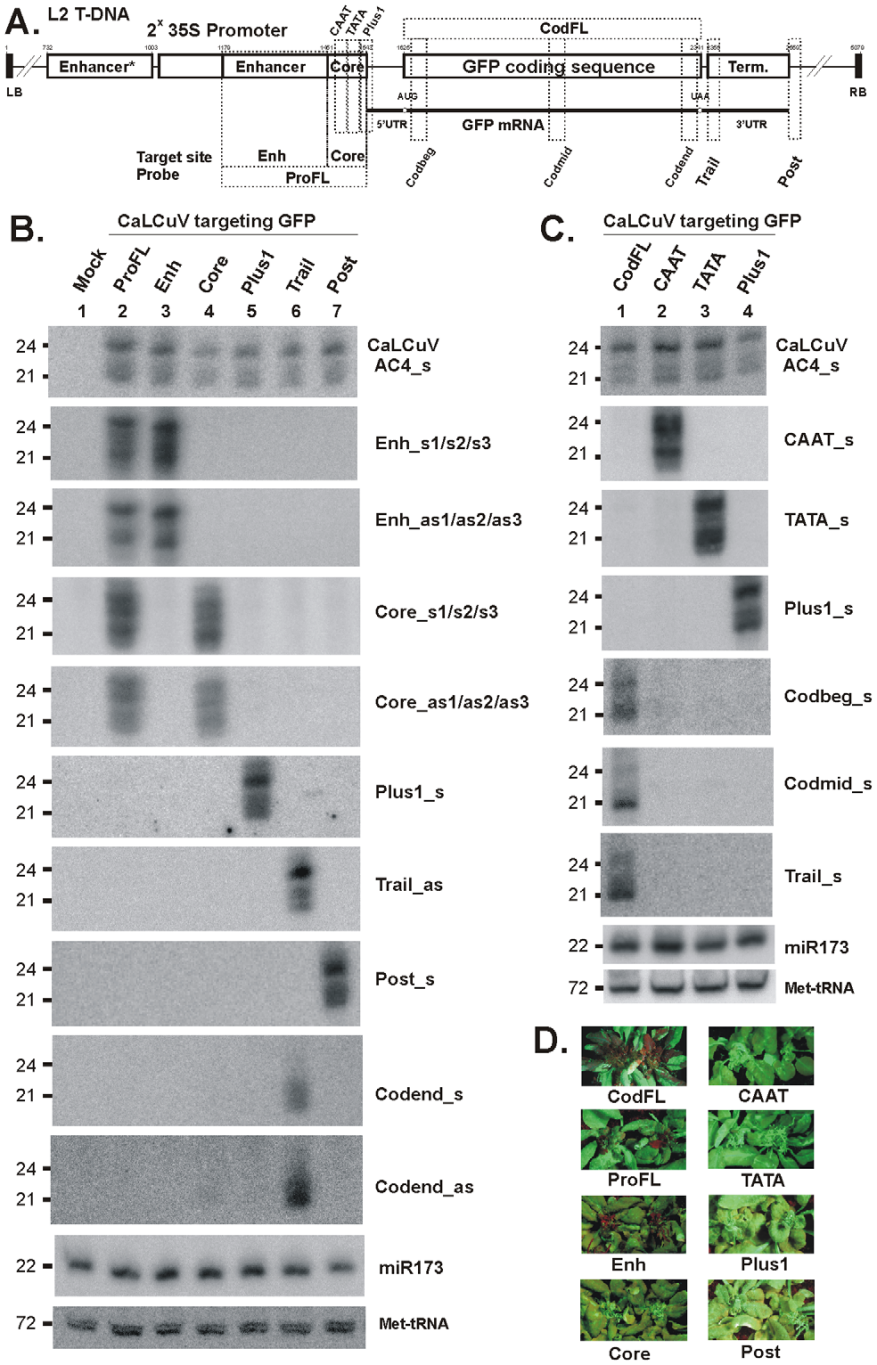
VIGS phenotypes and primary siRNA accumulation in L2 transgenic plants infected with CaLCuV::GFP viruses that target the *GFP* promoter and terminator elements. (**A**) The L2 T-DNA region containing the 35S-GFP transgene is shown schematically. Positions of the duplicated CaMV 35S enhancer (*Enh*) and core promoter (*Core*) elements (CAAT and TATA boxes and transcription start *Plus1*), the *GFP* mRNA elements (5′UTR, AUG and UAA codons and 3′UTR, and 35S terminator are indicated. Numbering is from the T-DNA left border (LB). The VIGS target sequences, inserted in the CaLCuV::GFP viruses *ProFL, Enh, CAAT, TATA, Plus1, CodFL, Trail* and *Post* are indicated with dotted boxes; (**B**) and (**C**) Blot hybridization analysis of total RNA isolated from L2 plants infected with the above viruses. The two blots were successively hybridized with short DNA probes specific for CaLCuV *AC4* gene (AC4_s) and the 35S::GFP transgene sequences inserted in CaLCuV::GFP viruses and *Arabidopsis* miR173 and Met-tRNA (the latter two serve as loading control). (**D**) Pictures under UV light of L2 transgenic plans infected with the CaLCuV::GFP viruses (names indicated).

**Figure 8:**
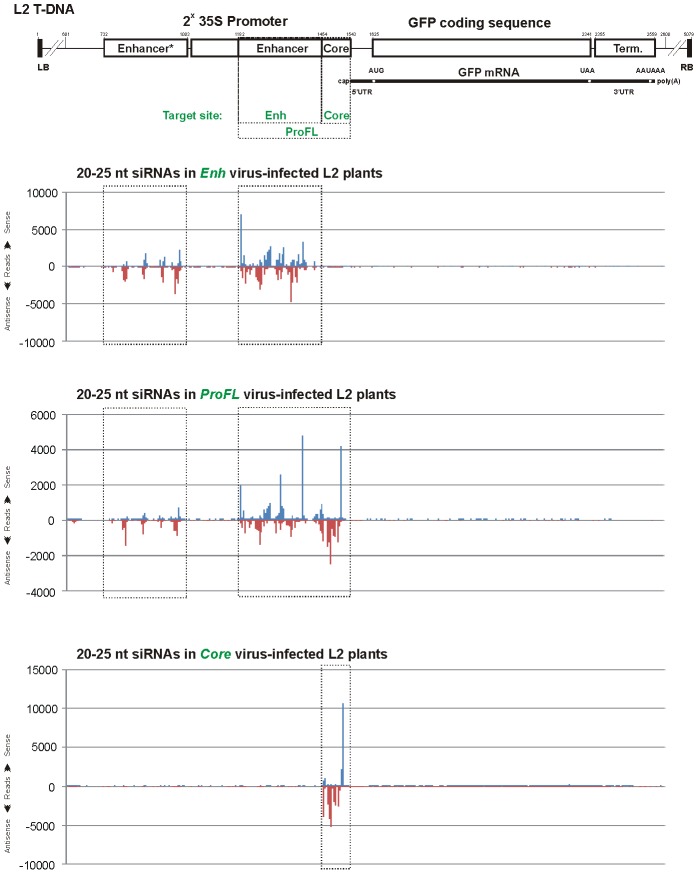
Maps of primary siRNAs accumulating in L2 transgenic plants infected with CaLCuV::GFP viruses that target the *GFP* promoter elements. The graphs plot the number of 20–25 nt vsRNA reads at each nucleotide position of the L2 T-DNA-based 35S::GFP transgene; Bars above the axis represent sense reads starting at each respective position; those below represent antisense reads ending at the respective position (Table S5). The 35S-*GFP* transgene is shown schematically above the graphs. Positions of the duplicated 35S enhancer and core promoter, *GFP* mRNA elements and 35S terminator are indicated. Numbering is from the T-DNA left border (LB). The VIGS target sequences inserted in the CaLCuV::GFP viruses *CodFL, Enh* and *Core* are indicated with dotted boxes. Note that the duplicated 35S promoter sequences Enhancer* and Enhancer (each 273 nt long) share 94% nucleotide identity, since they originate from two different strains of CaMV. Therefore, primary siRNA reads are unequally distributed between the two VIGS target regions.

Contrary to what we observed for the transcribed region, targeting of the *GFP* non-transcribed regions with short sequences inserted into the CaLCuV VIGS vector did not lead to *GFP* silencing or secondary siRNA production in systemically-infected L2 plants (Figure 7 and Figure S4). The 30-bp sequences which surround the 35S core promoter elements including the CAAT and TATA boxes (‘*CAAT*’ and ‘*TATA*’) and the transcription start site (‘*Plus1*’), or sequences that occur in a distal region of the 35S enhancer (‘*EnhSh*’) and just downstream of the mRNA processing/poly(A) addition site (‘*Post*’) gave rise to abundant siRNAs of the three major classes but no secondary siRNAs were detected outside of the target sequence. Furthermore, insertion of the 90-bp 35S core promoter region (‘*Core*’) did not result in *GFP* silencing or secondary siRNA production, despite abundant primary siRNAs targeting this region. However, insertions of the entire 35S enhancer region of 272 bp (‘*Enh*’) or the full-length promoter of 382 bp (‘*ProFL*’) resulted in systemic *GFP* silencing. But also in these two cases no secondary siRNAs were detected outside of the target region (Figure 7). These findings were confirmed by Illumina sequencing of sRNAs from L2 plants systemically infected with *Core, Enh* and *ProFL* viruses (Figures 8 and Figure S6; Table S5 and Table S6). In addition, the deep sequencing revealed that, besides extremely low levels of secondary siRNA accumulation outside of the target region, there appear to be almost no secondary siRNA amplification within the target region. Thus, the duplicated 273-bp Enhancer* region shares 94% nucleotide identity with the target Enhancer region, since these sequences originate from two different strains of CaMV, and we found only negligible numbers of reads in the three stretches of the Enhancer* sequence that have mismatches to corresponding stretches of the Enhancer sequence (Figure 8; Table S5, see positions 760–781, 803–837 and 869–905).

Taken together, we conclude that production of abundant secondary siRNAs can be triggered by primary virus-derived siRNAs that target *GFP* mRNA. Hence, CaLCuV infection does not block amplification of secondary siRNAs likely mediated by RDR activities (see below). This is also supported by our blot hybridization analysis showing that accumulation of RDR6-dependent tasiRNAs is not significantly affected by CaLCuV infection (Figure S2; siR255). Both primary (virus-derived) and secondary siRNAs correlate with efficient *GFP* silencing. However, targeting of the non-transcribed, 35S enhancer region by primary siRNAs induces efficient *GFP* silencing without any substantial production of secondary siRNAs. Hence, secondary siRNAs do not appear to be necessary for silencing *GFP* transgene, at least at the transcriptional level. Previously, transcriptional VIGS through targeting the 35S promoter region of 35S::*GFP* transgene was observed but its dependence on primary or secondary siRNAs was not tested in that case [58].

### *GFP* secondary siRNAs are RDR6-dependent

To investigate genetic requirements for the biogenesis of *GFP* secondary siRNAs, the L2 transgenic line was crossed with the Col-0 mutant lines carrying point mutations in *RDR6* (*rdr6*-*14*; [59]) and *DCL4* (*dcl4-2*; [60]). The resulting homozygous mutant lines L2 x *rdr6* and L2 x *dcl4* expressed high levels of GFP, similar to those of the parental L2 plants (not shown).

**Figure 9:**
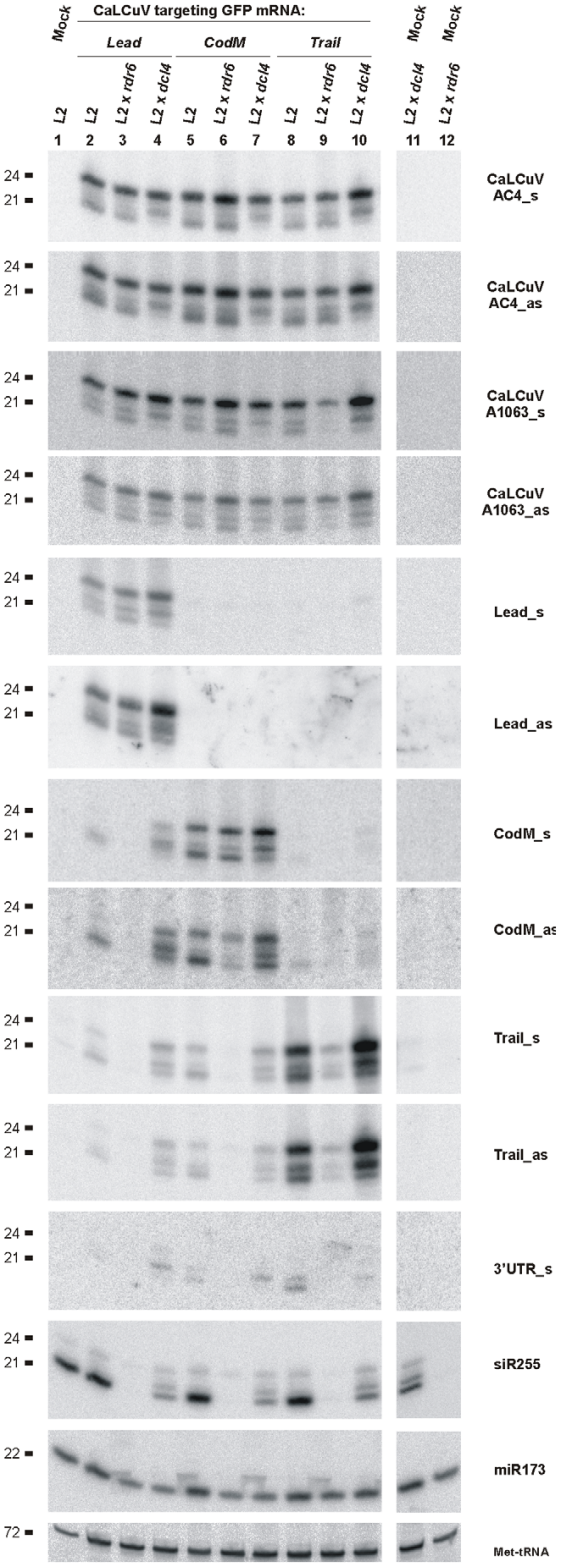
Genetic requirements for primary and secondary siRNA accumulation in L2 transgenic plants. Blot hybridization analysis of total RNA isolated from L2, L2 x *rdr6* and L2 x *dcl4* plants infected with CaLCuV::GFP viruses *Lead, CodM* and *Trail*. The blot was successively hybridized with short DNA probes specific for CaLCuV genes AC4 (AC4_s and AC4_as) and AV1 (A1063_s and A1063_as), 35S::GFP transgene sequences inserted in the CaLCuV::GFP viruses (*Lead, CodM, Trail*), *GFP* mRNA 3′UTR non-target sequence (3′UTR_s) and *Arabidopsis* miR173 and Met-tRNA (the latter two serve as loading control).

Systemic infection of L2 x *rdr6* and L2 x *dcl4* plants with the recombinant viruses *Lead, CodM* and *Trail* resulted in *GFP* silencing in all cases, except L2 x *rdr6* plants infected with the *Lead* virus. Consistent with our findings for wild-type CaLCuV (Figure S2) and CaLCuV::Chl ([7]; Figure S2), blot hybridization analysis revealed that the biogenesis of 21, 22 and 24 nt vsRNAs derived from the *AC4* ORF region of the three recombinant viruses was not affected in L2 x *rdr6* plants lacking RDR6 (Figure 9). By contrast, probes specific for the target transgene revealed a major contribution of RDR6 in secondary siRNA production. In fact, production of secondary siRNAs of all size-classes outside of the target region was nearly abolished in L2 x *rdr6* plants infected with *Lead, CodM* and *Trail* viruses (Figure 9). For the latter two viruses, accumulation of siRNAs from the insert/target sequence was also reduced: interestingly, the reduced accumulation was observed for siRNAs of sense but not antisense polarity in *CodM* virus, while siRNAs of both polarities were strongly reduced in *Trail* virus. By contrast, accumulation of siRNAs from the *Lead* insert/target sequence was not altered in L2 x *rdr6* plants infected with *Lead* virus (Figure 9). We conclude that RDR6-independent primary vsRNAs represent the majority of siRNAs derived from the *Lead* sequence, whereas the *CodM* and *Trail* sequences also spawn RDR6-dependent secondary siRNAs in addition to primary vsRNAs. These secondary siRNAs could potentially be produced from the transgene and/or the viral insert. We therefore used the probes specific to the viral sequence located just downstream of the insert (CbA1063_s and CbA1063_as), i.e. present in the chimeric rightward viral transcript. The results revealed that, in the case of *Lead* and *CodM* viruses, RDR6 is not involved in production of vsRNAs from this region (Figure 9). Thus, the contribution of RDR6 to siRNA production from the *CodM* insert/target sequence of antisense polarity can be explained by RDR6-dependent siRNA production from the target gene rather than the chimeric virus. However, accumulation of vsRNAs derived from the chimeric transcript region of *Trail* virus was substantially reduced (24-nt) or nearly abolished (21-nt and 22-nt) in L2 x *rdr6* plants. This indicates that, in addition to the transgenic mRNA, the chimeric viral transcript can also be used for RDR6-dependent production of secondary siRNAs. But the insert sequence itself appears to regulate relative contribution of RDR6. Notably, the *ChlI* insert sequence does not make the chimeric viral transcript prone to RDR6-dependent vsRNA production (Figure S2). It remains to be further investigated why the *Trail* (but not *Lead*, *CodM or ChlI*) sequence makes the viral chimeric transcript prone to RDR6-dependent amplification of secondary siRNAs. Interestingly, this sequence originates from the CaMV terminator/leader region and contains two stretches of AG-repeats (Protocol S1).

It is puzzling that, in the absence of RDR6-dependent secondary siRNAs in L2 x *rdr6* plants, the *GFP* silencing is efficiently triggered by *CodM* and *Trail* viruses but not by *Lead* virus. We speculate that *GFP* mRNA cleaved by primary siRNAs within its 5′UTR can still be translated, unless it enters the RDR6 pathway converting the coding and 3′UTR sequences to secondary siRNAs. By contrast, primary siRNA-directed cleavage within the coding sequence or 3′UTR would block productive translation and could therefore be sufficient for *GFP* silencing.

In L2 x *dcl4* plants, we detected reduced accumulation of 21-nt primary siRNAs from the viral *AC4* region and 21-nt primary and secondary siRNAs from the *GFP* sequences. Unexpectedly, accumulation of 22-nt and 24-nt primary and secondary siRNAs was increased: this increase was more prominent for secondary *GFP* siRNAs (Figure 9). This resembles the shift in the profile of RDR6-dependent 21-nt tasiRNAs in this particular mutant background ([60]; Figure 9, see tasiRNA siR255). Thus, a mutated DCL4 protein expressed from the *dcl4-2* allele appears to promote processing of RDR6-dependent dsRNAs by alternate DCLs that generate longer siRNAs (i.e. DCL2 and DCL3).

Taken together, our findings confirm a major role of DCL4 in processing 21-nt secondary siRNAs from RDR6-dependent dsRNA precursors derived from the transgene and 21-nt primary vsRNAs from RDR6-independent viral dsRNA precursors. In addition, our results reveal that RDR6-dependent dsRNA can be efficiently processed by alternate DCL activities if the DCL4 protein is mutated by an amino acid substitution in the helicase domain. These alternate DCLs produce primary and secondary siRNAs which are equally potent in *GFP* silencing, since we did not observe any substantial difference in systemic silencing phenotypes between wild-type and *dcl4-2* plants infected with any of the recombinant viruses. This is in line with our previous findings for CaLCuV::Chl-derived primary vsRNAs of distinct classes produced in single, double and triple *dcl* mutant plants, which could efficiently knockdown *ChlI* mRNA [7]. Previously, a major role of DCL2 was established for production of secondary siRNAs in a transgene targeted by primary siRNAs from another transgene [11]. Here, in addition to DCL2, we find the apparent involvement of DCL3 which normally generates 24-nt nuclear siRNAs in secondary siRNA production. Thus, a fraction of dsRNA precursors of the *GFP* transgene-derived secondary siRNAs might be localized in the nucleus. Alternatively, a fraction of DCL3 protein might also be cytoplasmic.

### Concluding remarks

Secondary siRNAs are involved in various silencing pathways in plants, fungi and some animals. In *C. elegans*, RDR-dependent amplification of secondary siRNAs appears to reinforce silencing triggered by primary siRNAs which are processed by dicer from endogenous or exogenous dsRNA [61], [62]. In plants, some of the endogenous mRNAs targeted by miRNAs spawn RDR6-dependent secondary RNAs, a contribution of which to miRNA-directed gene silencing is not fully clarified [14], [15]. In most cases, plant miRNA-directed cleavage or translational repression is sufficient for robust gene silencing without production of secondary siRNAs [14]. Likewise, most plant mRNAs silenced by transgene- or virus-derived primary siRNAs do not spawn secondary siRNAs. This suggests that plant mRNAs could have evolved to be poor templates for RDR activity. Our study supports this notion by demonstrating that *Arabidopsis ChlI* and *ChlI-2* mRNAs that undergo robust VIGS spawn only small amounts of secondary siRNAs. Furthermore, we demonstrate that geminiviral mRNAs, which can potentially be targeted by highly abundant vsRNAs of antisense polarity (Figure 2), are not templates for RDR1-, RDR2-, or RDR6-dependent siRNA amplification. By contrast, the transgenic *GFP* mRNA targeted by primary viral siRNAs spawns massive amounts of secondary siRNAs whose production requires RDR6. Our findings suggest that some aberrant feature(s) of the transgenic *GFP* mRNA possessing non-self UTR sequences may attract RDR6 activity. Notably, the involvement of RDR6 and RDR1 in production of viral siRNAs in RNA virus-infected plants was revealed only by using the mutant RNA viruses carrying deletions or point mutations in viral silencing suppressor genes: unlike wild-type RNA, the mutated viral RNA spawned RDR-dependent vsRNAs. What makes mutant/chimeric viral mRNAs and transgenic mRNAs good templates for RDR activity remains unclear. One possibility is that viral and plant mRNAs could have evolved primary sequence or secondary structure elements that block RDR activity. Such elements may accidentally be disrupted by mutations in the suppressor-deficient RNA viruses. Likewise, transgene transcripts might lack some of the naturally evolved sequence or structure elements.

Our findings suggest that the precursors of geminiviral siRNAs are most likely produced by Pol II-mediated bidirectional readthrough transcription in both sense and antisense orientations on the circular viral DNA. Such transcripts (or their degradation products) can potentially pair viral mRNAs and thus form perfect dsRNAs to be processed by multiple DCLs into vsRNAs. Readthrough transcription far beyond a poly(A) signal is a known property of Pol II. In pararetroviruses, it represents an obligatory mechanism by which a pregenomic RNA covering the entire circular genome is generated. The poly(A) signal of plant pararetroviruses is located at a relatively short distance (e.g. 180 bp in CaMV) downstream of the pregenomic RNA promoter: this allows efficient readthrough transcription at the first encounter by the Pol II complex and termination of transcription at the second encounter [63], [64]. Thus, substantial readthrough transcription can also be expected in geminiviruses which possess relatively short transcription units. Evidence for the existence of readthrough transcripts was obtained earlier for a related geminivirus [34] and is also provided here by deep sequencing showing that vsRNAs of both sense and antisense polarities densely tile along the entire CaLCuV genome including “non-transcribed” intergenic region of both DNA-A and DNA-B. Pol II readthrough transcription downstream of a canonical poly(A) signal of the endogenous *A. thaliana* gene *FCA* was recently shown to be repressed by a DCL4-dependent mechanism [12]. In a *dcl4* mutant, the increased transcriptional readthrough far beyond the *FCA* poly(A) signal triggered silencing of a transgene containing the same 3′ region. Notably, the transgene silencing was caused by RDR6-dependent production of very abundant 22-nt siRNAs by DCL2 and less abundant 24-nt siRNAs by DCL3. This siRNA pattern resembles the pattern of *GFP* transgene-derived secondary siRNAs that we observed in L2 x *dcl4* plants (Figure 9). Also in line with our observations, robust siRNA-directed silencing of the transgene and *FCA* did not spread to a converging gene that overlaps with the *FCA* readthrough transcript [12], further supporting the notion that most endogenous genes are not prone to RDR6-dependent transitivity.

## Materials and Methods

### Plant mutants and virus infection

*Arabidopsis thaliana* wild-type (Col-0) and *rdr2/6*, *rdr1/2/6* and *dcl1/2/3/4* mutant lines used in this study, their growth conditions and infection with wild-type CaLCuV (the DNA-A clone ‘CLCV-A dimer’ [33] and the DNA-B clone pCPCbLCVB.002 [50]) and CaLCuV::Chl (pMTCbLCVA::CH42 and pCPCbLCVB.002 [50]) using biolistic delivery of viral DNA have been described earlier [7], [8]. Using the same protocols, L2 transgenic plants (Line 2; [54]) were grown and inoculated with CaLCuV::GFP viruses.

L2 plants [54] were crossed with the *dcl4-2* and *rdr6-14* mutants [59], [60]. L2 homozygosity was determined by PCR in the F2 populations using 5′-TTGCTGCAACTCTCTCAGGGCC-3′ and 5′-GATAAATGTGGAGGAGAAGACTGCC-3′ for detecting the presence of the T-DNA and 5′-ACACTCTCTCTCCTTCATTTTCA-3′ and 5′-TCTGCAACACTCTGTCATTGG-3′ for detecting the absence of intact genomic region. RDR6-14 homozygosity was determined by visual observation of the typical epinastic leaf phenotype of the *rdr6* mutants and was further confirmed using a dCAPS marker consisting of *NcoI* digestion of the PCR product obtained using 5′-AAGATTTGATCCCTGAGcCAT-3′ and 5′-GTTCGCCTTGTTTCTTGCTT-3′. DCL4-2 homozygosity was determined by the typical epinastic leaf phenotype of the *dcl4* mutants. Homozygosity for L2 and the respective mutations were confirmed in F3 plants following the same procedures.

### Construction of recombinant viruses

The CaLCuV::GFP viruses *EnhSh, CAAT, TATA, Plus1, Lead, Start, CodB, CodM, CodE, Stop, Trail, PolyA* and *Post* were generated by cloning preannealed sense and antisense oligonucleotides (listed in Protocol S1) into *XbaI* and *XhoI* sites of the CaLCuV VIGS vector pCPCbLCVA.007 [50]. The CaLCuV::GFP viruses *Enh, Core* and *ProFL* were generated by subcloning into *XbaI* and *XhoI* sites of pCPCbLCVA.007 the corresponding regions of the L2 T-DNA 35S promoter using PCR with primers listed in Protocol S1 on total DNA isolated from L2 transgenic plants. In all the above derivatives of the CaLCuV VIGS vector the insert sequences are in antisense orientation with respect of the *AV1* gene promoter.

### sRNA analysis

For both blot hybridization and Illumina deep-sequencing, aerial tissues of three virus-infected (or mock-inoculated) plants were harvested one month post-inoculation and pooled for total RNA preparation using a Trizol method [7]. sRNA blot hybridization analysis was performed as in Blevins et al. [7] using short DNA oligonucleotide probes listed in Protocol S1. cDNA libraries of the 19–30 nt RNA fraction of total RNA samples were prepared as we described previously [8]. The high-coverage libraries of wild-type CaLCuV were sequenced on an Illumina Genome Analyzer (GA) *Hi-Seq 2000* using a *TruSeq v5* kit, while the low coverage libraries on a GA-II using *Chrysalis v2*. The libraries of CaLCuV::Chl and CaLCuV::GFP viruses were sequenced on a GA-IIx using *Chrysalis v4* and *TruSeq v5*, respectively. After trimming the adaptor sequences, the datasets of reads were mapped to the reference genomes of *Arabidopsis thaliana* Col-0 (TAIR9), CaLCuV (U65529.2 for DNA-A and U65530.2 for DNA-B) and other references using a Burrows-Wheeler Alignment Tool (BWA version 0.5.9) [65] with zero mismatches to the reference sequence. The reference sequences of CaLCuV DNA-A and its derivatives, CaLCuV DNA-B, L2 T-DNA and *ChlI*/CH42 and *ChlI-2* genomic loci are given in Protocol S1. Reads mapping to several positions on the references were attributed at random to one of them. To account for the circular virus genome the first 50 bases of the viral sequence were added to its 3′-end. For each reference genome/sequence and each sRNA size-class (20 to 25 nt), we counted total number of reads, reads in forward and reverse orientation, and reads starting with A, C, G and T (Table S1). In the single-base resolution maps of 20, 21, 22, 23, 24 and 25 nt vsRNA (Tables S2, S3, S4, S5, S6 and S7), for each position on the sequence (starting from the 5′ end of the reference sequence), the number of matches starting at this position in forward (first base of the read) and reverse (last base of the read) orientation for each read length is given. Note that the reads mapped to the last 50 bases of the extended viral sequence were added to the reads mapped to the first 50 bases.

### Analysis of long viral RNA and DNA by blot hybridization

The detailed protocol for high-resolution analysis of long RNA using total RNA and 5% PAGE followed by blot hybridization was described previously [30]. To detect the viral mRNAs AV1, AC2/AC3, BV1 and BC1 (Figure 3A), the membrane was successively hybridized with mixtures of DNA oligonucleotides complementary to each given mRNA (for sequences, see Protocol S1).

Southern blot analysis was performed as in [66]. In short, total DNA from the plants were extracted by CTAB-based protocol. Five µg of total DNA was electrophoresed in 1% agarose gel prepared in 1× Tris-sodium acetate-EDTA buffer. Full-length linear DNA of CaLCuV was loaded as a positive control for Southern hybridization. After EtBr staining, the DNA in the gel was alkali-denatured and transferred to the Hybond N+ nylon membrane (GE healthcare lifesciences). PCR fragments of DNA-A (900 bp obtained with the primers Cb_AV1_qPCR_s and Cb_AC3_qPCR_as) and DNA-B (862 bp Cb_BV1_qPCR_s and Cb_BC1_qPCR_as), which do not contain the common region of the virus, were labeled with [α-32P]dCTP using Rediprime II DNA labeling system (GE healthcare lifesciences) and used as probes. Hybridization with the labeled probe was performed at 65°C for 16–20 hours using PerfectHyb Plus Hybridization Buffer (Sigma-Aldrich) and the membrane was washed thrice at 65°C with 2× SSC/0.5% SDS. The signal was detected after 5 days exposure to a phosphor screen using a Molecular Imager (Typhoon FLA 7000, GE healthcare lifesciences).

### Real time PCR

Relative accumulation of polyadenylated viral mRNAs and total viral DNA in wild type versus *rdr1/2/6* (Figure 3D) was measured using real time PCR as in [8]. For polyadenylated RNA, cDNA was synthesized from 5 µg of total RNA using 100 pmoles of oligo d(T)16 primer. The RNA-primer mixture was heated to 70°C for 10 min and chilled on ice for 5 min. 4 µl of 5× first-strand synthesis buffer (250 mM Tris-HCl [pH 8.3], 375 mM KCl, 15 mM MgCl2, 0.1 M DTT), 2 µl 0.1 M DTT, 1 µl 10 mM deoxynucleoside triphosphate mix and 1 µl (200 U) of Superscript III reverse transcriptase (Invitrogen) were added and incubated at 50°C for 60 min. The reaction was stopped by heating the mixture to 95°C for 5 min. 2 µl of the 10 times diluted reverse transcription reaction mix or 2 µl of total DNA (2 ng) were taken for PCR in LightCycler 480 Real-Time PCR System (Roche applied sciences) using FastStart Universal SYBR Green Master (Rox) mix (Roche) and primers designed using Beacon designer 2 software (PREMIER Biosoft International). PCR primers specific for viral DNAs A and B and each viral mRNA as well as internal controls (18S rDNA and *ACT2* mRNA) are given in Protocol S1. Cycling parameters were 95°C for 10 min, followed by 45 cycles: 95°C for 10 s, 56°C for 10 s, 72°C for 20 s. Amplification efficiency of primers was determined by means of a calibration curve (Ct value vs. log of input cDNA/DNA) prepared in triplicate. The Ct values obtained for viral genes were normalized with internal control values and the delta Ct values were obtained. The normalized values for CaLuCV-infected wild type Col-0 were set to 1. To quantify the L2 GFP mRNA levels, poly-dT cDNAs were made as described above. Real-time PCR was performed in 96-well titer plates on an ABI PRISM 7000 SDS apparatus with SYBR GREEN PCR Master Mix (ABI) following manufacturers' recommendations (95°C for 5 min., followed by 40 cycles: 95°C for 30 s, 60°C for 45 s). Primers are given in Protocol S1. Uncertainties were propagated from standard errors for triplicate measurements of cDNA pools (derived from column-purified RNA of 3–4 plants).

## Supporting Information

Figure S1 **Maps of 21, 22 and 24 nt vsRNAs from CaLCuV-infected wild type (Col-0) and** ***rdr1/2/6*** **triple mutant plants at single-nucleotide resolution.** The graphs plot the number of 21-nt, 22-nt, or 24-nt vsRNA reads at each nucleotide position of the 2583 bp DNA-A (**A**) and the 2513 bp DNA-B (**B**); Bars above the axis represent sense reads starting at each respective position; those below represent antisense reads ending at the respective position (Tables S2 and S3). The genome organizations of DNA-A and DNA-B are shown schematically above the graphs, with leftward (AC1, AC4, AC2, AC3 and BC1) and rightward (AV1 and BV1) ORFs and common region (CR) indicated.
(PDF)

Figure S2 **Validation of vsRNA deep-sequencing data and genetic requirements for vsRNA biogenesis.** Total RNA isolated from CaLCuV wild type (wt) virus- or CaLCuV::Chl-infected *Arabidopsis* wt (Col-0) plants and various mutants (*rdr2, rdr6, rdr2/6, rdr1/2/6* and *dcl1/2/3/4-caf*; described in Blevins et al, 2006) was analyzed by RNA blot hybridization using 15% PAGE. Membranes were successively hybridized with CaLCuV DNA-A (**A**) and CaLCuV DNA-B (**B**) derived DNA oligonucleotide probes (for sequences, see Protocol S1) or probes specific the endogenous *Arabidopsis* small RNAs (**C**) 22 nt miR173, 21 nt siR255 and 24 nt siR1003. The probes Chl_s and Chl_as in panel A are specific for the *ChlI* gene segment inserted in CaLCuV::Chl DNA-A. EtBr staining of total RNA is shown as loading control. The sizes are indicated on each scan.
(PDF)

Figure S3 **Viral and target gene siRNAs in CaLCuV::Chl virus-infected wild type (Col-0) plants.** (**A**) The 1961 bp *ChlI-2* genomic locus is shown schematically; numbering starts from the transcription start site. The VIGS target region is highlighted in grey, with the two stretches of >20 nts in length which are identical in *ChlI* and *ChlI-2* shown in red. The graph plots the number of 20–25 nt siRNA reads at each nucleotide position of the *ChlI-2* gene; Bars above the axis represent sense reads starting at each respective position; those below represent antisense reads ending at the respective position (Table S4). (**B**) Alignment of the *ChlI and ChlI-2* sequences containing the VIGS target region is shown below the graph; (**C**) Virus-derived siRNAs. The graphs plot the number of 20–25 nt, 21-nt, 22-nt, or 24-nt vsRNA reads at each nucleotide position of the 2298 bp CaLCuV::Chl DNA-A. Bars above the axis represent sense reads starting at each respective position; those below represent antisense reads ending at the respective position (Tables S4). The genome organization of CaLCuV::Chl DNA is shown schematically above the graphs, with leftward (AC1, AC4, AC2, AC3 and BC1) ORFs and the rightward AV1::Chl chimeric gene and the common region (CR) indicated. The 353 bp *ChlI* gene segment inserted in the multiple cloning site (MCS) of the CaLCuV VIGS vector is highlighted in grey.
(PDF)

Figure S4 **VIGS phenotypes and accumulation of primary and secondary siRNAs in L2** ***GFP*** **transgenic plants infected with CaLCuV::GFP viruses.** (**A**) The L2 T-DNA region containing the 35S-GFP transgene is shown schematically. Positions of the duplicated CaMV 35S enhancer and core promoter elements, *GFP* mRNA elements including 5′UTR, translation start (AUG) and stop (UAA) codons and 3′UTR with poly(A) signal (AAUAAA), and 35S terminator sequences are indicated. Numbering is from the T-DNA left border (LB). The VIGS target sequences, inserted in the CaLCuV::GFP viruses *EnhSh*, *CodM, CodE* and *CodFL* are indicated with dotted boxes. (**B**) Pictures under UV light of the L2 transgenic plant infected with the *CodFL* virus at 7, 12, 19, 26 and 33 days post-inoculation (dpi) and of the same plant at 40 dpi under UV and day light. Below are pictures under UV light of L2 plants infected with the CaLCuV empty vector and its derivatives *EnhSh*, *CodM* and *CodE*. Sampling of infected tissues of lower leaves (LL) and upper leaves (UL) for RNA preparation was performed as indicated on the left image. (**C**) Blot hybridization analysis of total RNA isolated from plants shown in Panel B. The blot was successively hybridized with short DNA probes specific for 35S::GFP transgene sequences inserted in the CaLCuV::GFP viruses *EnhSh*, *CodM* and *CodE* and for the *GFP* mRNA 3′UTR non-target sequence (3′UTR). EtBr staining serves as loading control. (**D**) Real time quantitative RT-PCR (qPCR) analysis of *GFP* mRNA accumulation in upper leaves of L2 plants infected with infected with the CaLCuV empty vector and its derivatives *EnhSh*, *CodM, CodE* (shown in Panel B). Total RNA from non-transgenic wild type *Arabidopsis* (Col-0) was used as a negative control.
(PDF)

Figure S5 **Maps of primary and secondary siRNAs accumulating in L2 transgenic plants infected with CaLCuV::GFP viruses that target the** ***GFP*** **transcribed region.** The graphs plot the number of 21-nt, 22-nt and 24-nt vsRNA reads at each nucleotide position of the L2 T-DNA-based 35S::GFP transgene in L2 transgenic plants infected with the CaLCuV::GFP viruses *Lead* (**A**), *CodM* (**B**), *Trail* (**C**), or *PolyA* (**D**). Bars above the axis represent sense reads starting at each respective position; those below represent antisense reads ending at the respective position (Table S5). The 35S-GFP transgene is shown schematically above the graphs. Positions of the duplicated 35S enhancer and core promoter, *GFP* mRNA elements and 35S terminator are indicated. Numbering is from the T-DNA left border (LB). The VIGS target sequences inserted in the CaLCuV::GFP viruses *Lead, CodM, Trail* or *polyA* are indicated with dotted boxes.
(PDF)

Figure S6 **Maps of primary siRNAs accumulating in L2 transgenic plants infected with CaLCuV::GFP viruses that target the** ***GFP*** **promoter elements.** The graphs plot the number of 21-nt, 22-nt and 24-nt vsRNA reads at each nucleotide position of the L2 T-DNA-based 35S::GFP transgene in L2 transgenic plants infected with the CaLCuV::GFP viruses *Enh* (**A**), *ProFL* (**B**) or *Core* (**C**). Bars above the axis represent sense reads starting at each respective position; those below represent antisense reads ending at the respective position (Table S5). The 35S-GFP transgene is shown schematically above the graphs. Positions of the duplicated 35S enhancer and core promoter, *GFP* mRNA elements and 35S terminator are indicated. Numbering is from the T-DNA left border (LB). The VIGS target sequences inserted in the CaLCuV::GFP viruses *Enh, CodFL* and *Core* are indicated with dotted boxes. Note that the duplicated 35S promoter sequences Enhancer* and Enhancer (each 273 nt long) share 94% nucleotide identity, since they originate from two different strains of CaMV. Therefore, primary siRNA reads are unequally distributed between the two VIGS target regions.
(PDF)

Protocol S1 **The file contains the list of DNA oligonucleotides probes for RNA and DNA blot hybridization, primers for subcloning of the 35S::GFP tarnsgene-derived sequences into CaLCuV VIGS vector and for real time PCR as well as Reference sequences used for bioinformatic analysis.**
(PDF)

Table S1 **Counts of viral and endogenous small RNAs in the Illumina small RNA deep-sequencing libraries for mock-inoculated and wild type CaLCuV-infected Col-0 and** ***rdr1/2/6*** **plants (S1A), mock inoculated and CaLCuV::Chl-infected Col-0 plants (S1B), mock inoculated and CaLCuV::** ***GFP-Pro-FL*** **-infected Col-0 plants (S1C), mock inoculated and CaLCuV::** ***GFP-Enh*** **-infected Col-0 plants (S1D), mock inoculated and CaLCuV::** ***GFP-Core*** **-infected Col-0 plants (S1E), mock inoculated and CaLCuV::** ***GFP-Lead*** **-infected Col-0 plants (S1F), mock inoculated and CaLCuV::** ***GFP-CodM*** **-infected Col-0 plants (S1G), mock inoculated and CaLCuV::** ***GFP-Trail*** **-infected Col-0 plants (S1H), and mock inoculated and CaLCuV::** ***GFP-PolyA*** **-infected Col-0 plants (S1I).**
(XLSX)

Table S2 **Single-base resolution maps of 20–25 nt DNA-A derived viral siRNAs in CaLCuV-infected wild type (Col-0) and** ***rdr1/2/6*** **triple mutant** ***Arabidopsis*** **plants.**
(XLS)

Table S3 **Single-base resolution maps of 20–25 nt DNA-B derived viral siRNAs in CaLCuV-infected wild type (Col-0) and** ***rdr1/2/6*** **triple mutant** ***Arabidopsis*** **plants.**
(XLS)

Table S4 **Single-base resolution maps of 20–25 nt** ***ChlI/CH-42*** **and** ***ChlI-2*** **derived siRNAs as well as CaLCuV::Chl virus-derived siRNAs in mock inoculated and CaLCuV::Chl-infected** ***Arabidopsis*** **plants.**
(XLS)

Table S5 **Single-base resolution maps of 20–25 nt** ***L2 GFP T-DNA*** **derived siRNAs in mock inoculated and CaLCuV::** ***GFP*** **virus (** ***ProFL, Enh, Core, Lead, CodM, Trail*** **, or** ***PolyA*** **)-infected** ***Arabidopsis*** **plants.**
(XLS)

Table S6 **Single-base resolution maps of 20–25 nt viral siRNAs in CaLCuV::** ***GFP*** **virus (** ***ProFL, Enh, Core, Lead, CodM, Trail*** **, or** ***PolyA*** **)-infected** ***Arabidopsis*** **plants.**
(XLSX)
