# Supplementary material for: Primary and Secondary siRNAs in Geminivirus-induced Gene Silencing
Source: PLoS Pathog. 2012 Sep 27;8(9):e1002941. doi: 10.1371/journal.ppat.1002941 (PMC3460622; doi:10.1371/journal.ppat.1002941)
Supplement: Figure S1 — Maps of 21, 22 and 24 nt vsRNAs from CaLCuV-infected wild type (Col-0) and rdr1/2/6 triple mutant plants at single-nucleotide resolution. The graphs plot the number of 21-nt, 22-nt, or 24-nt vsRNA reads at each nucleotide position of the 2583 bp DNA-A (A) and the 2513 bp DNA-B (B); Bars above the axis represent sense reads starting at each respective position; those below represent antisense reads ending at the respective position (Tables S2 and S3). The genome organizations of DNA-A and DNA-B are shown schematically above the graphs, with leftward (AC1, AC4, AC2, AC3 and BC1) and rightward (AV1 and BV1) ORFs and common region (CR) indicated. (PDF) [file ppat.1002941.s001.pdf]

Figure S1. Maps of 21, 22 and 24 nt vsRNAs from CaLCuV-infected wild type (Col-0) and *rdr1/2/6* triple mutant plants

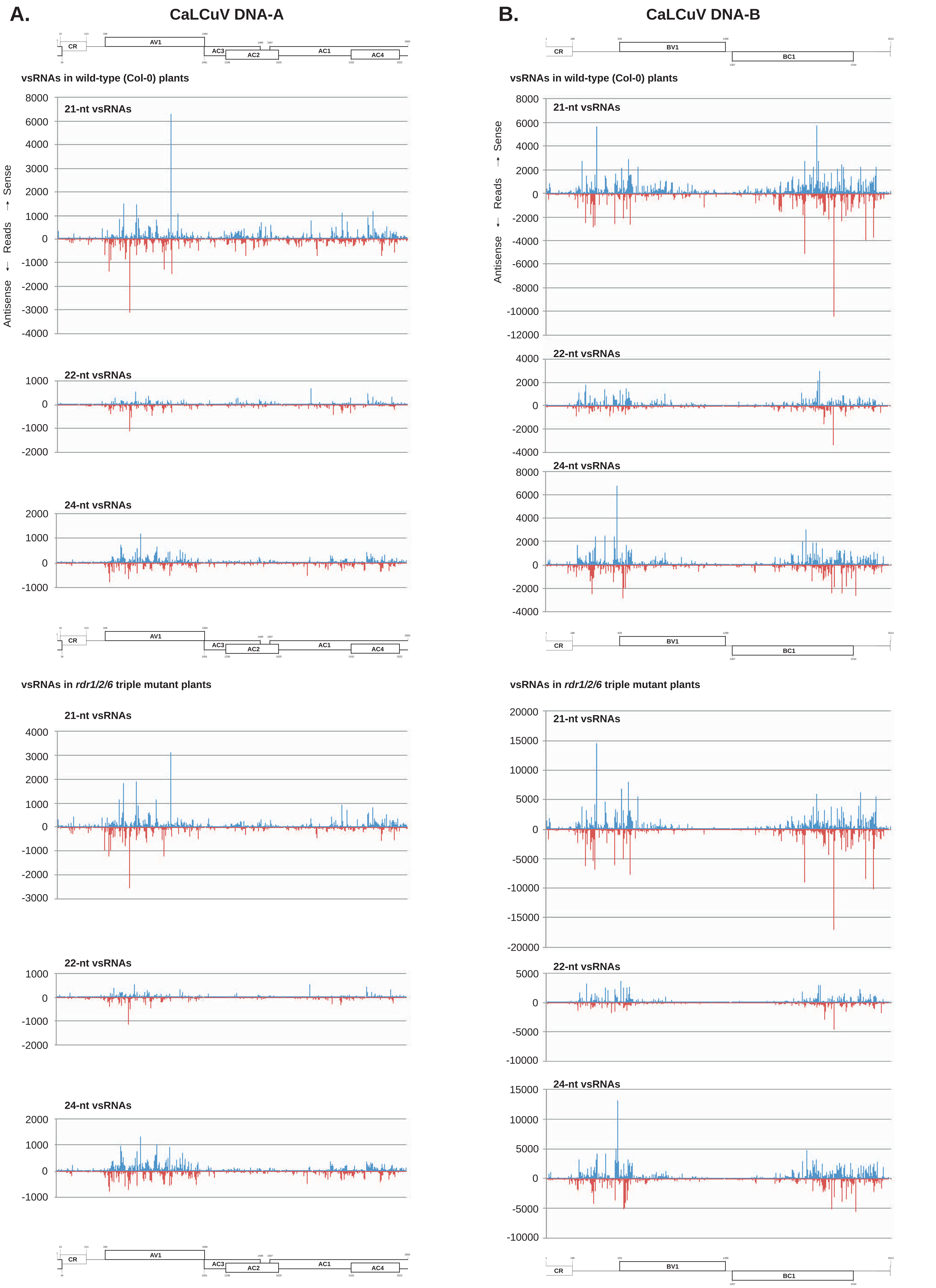

Figure S1. Maps of 21, 22 and 24 nt vsRNAs from CaLCuV-infected wild type (Col-0) and *rdr1/2/6* triple mutant plants at single-nucleotide resolution. The graphs plot the number of 21-nt, 22-nt, or 24-nt vsRNA reads at each nucleotide position of the 2583 bp DNA-A (A) and the 2513 bp DNA-B (B); Bars above the axis represent sense reads starting at each respective position; those below represent antisense reads ending at the respective position (Tables S2 and S3). The genome organizations of DNA-A and DNA-B are shown schematically above the graphs, with leftward (AC1, AC4, AC2, AC3 and BC1) and rightward (AV1 and BV1) ORFs and common region (CR) indicated.
