# Supplementary material for: Primary and Secondary siRNAs in Geminivirus-induced Gene Silencing
Source: PLoS Pathog. 2012 Sep 27;8(9):e1002941. doi: 10.1371/journal.ppat.1002941 (PMC3460622; doi:10.1371/journal.ppat.1002941)
Supplement: Figure S2 — Validation of vsRNA deep-sequencing data and genetic requirements for vsRNA biogenesis. Total RNA isolated from CaLCuV wild type (wt) virus- or CaLCuV::Chl-infected Arabidopsis wt (Col-0) plants and various mutants (rdr2, rdr6, rdr2/6, rdr1/2/6 and dcl1/2/3/4-caf; described in Blevins et al, 2006) was analyzed by RNA blot hybridization using 15% PAGE. Membranes were successively hybridized with CaLCuV DNA-A (A) and CaLCuV DNA-B (B) derived DNA oligonucleotide probes (for sequences, see Protocol S1) or probes specific the endogenous Arabidopsis small RNAs (C) 22 nt miR173, 21 nt siR255 and 24 nt siR1003. The probes Chl_s and Chl_as in panel A are specific for the ChlI gene segment inserted in CaLCuV::Chl DNA-A. EtBr staining of total RNA is shown as loading control. The sizes are indicated on each scan. (PDF) [file ppat.1002941.s002.pdf]

Figure S2. Validation of vsRNA deep-sequencing data and genetic requirements for vsRNA biogenesis

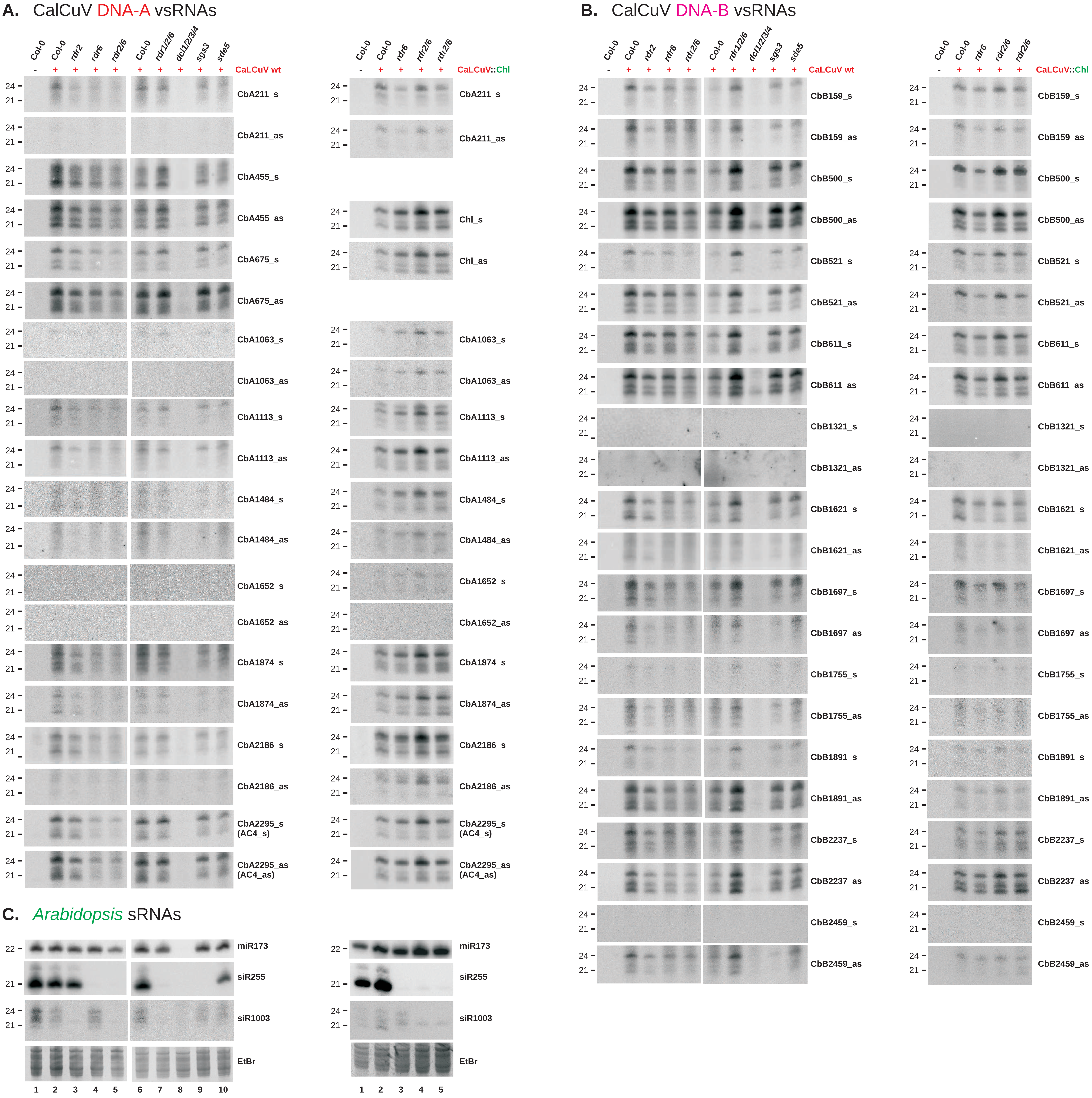

Figure S2. Validation of vsRNA deep-sequencing data and genetic requirements for vsRNA biogenesis. Total RNA isolated from CaLCuV wild type (wt) virus- or CaLCuV::ChI-infected Arabidopsis wt (Col-0) plants and various mutants (*rdr2, rdr6, rdr2/6, rdr1/2/6* and *dcl1/2/3/4*-caf; described in Blevins et al, 2006) was analyzed by RNA blot hybridization using 15% PAGE. Membranes were successively hybridized with CaLCuV DNA-A (A) and CaLCuV DNA-B (B) derived DNA oligonucleotide probes (for sequences, see Supplementary Methods) or probes specific the endogenous Arabidopsis small RNAs (C) 22 nt miR173, 21 nt siR255 and 24 nt siR1003. The probes ChI_s and ChI_as in panel A are specific for the ChII gene segment inserted in CaLCuV::ChI DNA-A. EtBr staining of total RNA is shown as loading control. The sizes are indicated on each scan.
