# Supplementary material for: Primary and Secondary siRNAs in Geminivirus-induced Gene Silencing
Source: PLoS Pathog. 2012 Sep 27;8(9):e1002941. doi: 10.1371/journal.ppat.1002941 (PMC3460622; doi:10.1371/journal.ppat.1002941)
Supplement: Figure S3 — Viral and target gene siRNAs in CaLCuV::Chl virus-infected wild type (Col-0) plants. (A) The 1961 bp ChlI-2 genomic locus is shown schematically; numbering starts from the transcription start site. The VIGS target region is highlighted in grey, with the two stretches of >20 nts in length which are identical in ChlI and ChlI-2 shown in red. The graph plots the number of 20–25 nt siRNA reads at each nucleotide position of the ChlI-2 gene; Bars above the axis represent sense reads starting at each respective position; those below represent antisense reads ending at the respective position (Table S4). (B) Alignment of the ChlI and ChlI-2 sequences containing the VIGS target region is shown below the graph; (C) Virus-derived siRNAs. The graphs plot the number of 20–25 nt, 21-nt, 22-nt, or 24-nt vsRNA reads at each nucleotide position of the 2298 bp CaLCuV::Chl DNA-A. Bars above the axis represent sense reads starting at each respective position; those below represent antisense reads ending at the respective position (Tables S4). The genome organization of CaLCuV::Chl DNA is shown schematically above the graphs, with leftward (AC1, AC4, AC2, AC3 and BC1) ORFs and the rightward AV1::Chl chimeric gene and the common region (CR) indicated. The 353 bp ChlI gene segment inserted in the multiple cloning site (MCS) of the CaLCuV VIGS vector is highlighted in grey. (PDF) [file ppat.1002941.s003.pdf]

Figure S3. Viral and target gene siRNAs in CaLCuV::ChI virus-infected wild type (Col-0) plants

A. *Chlorata I-2* genomic locus

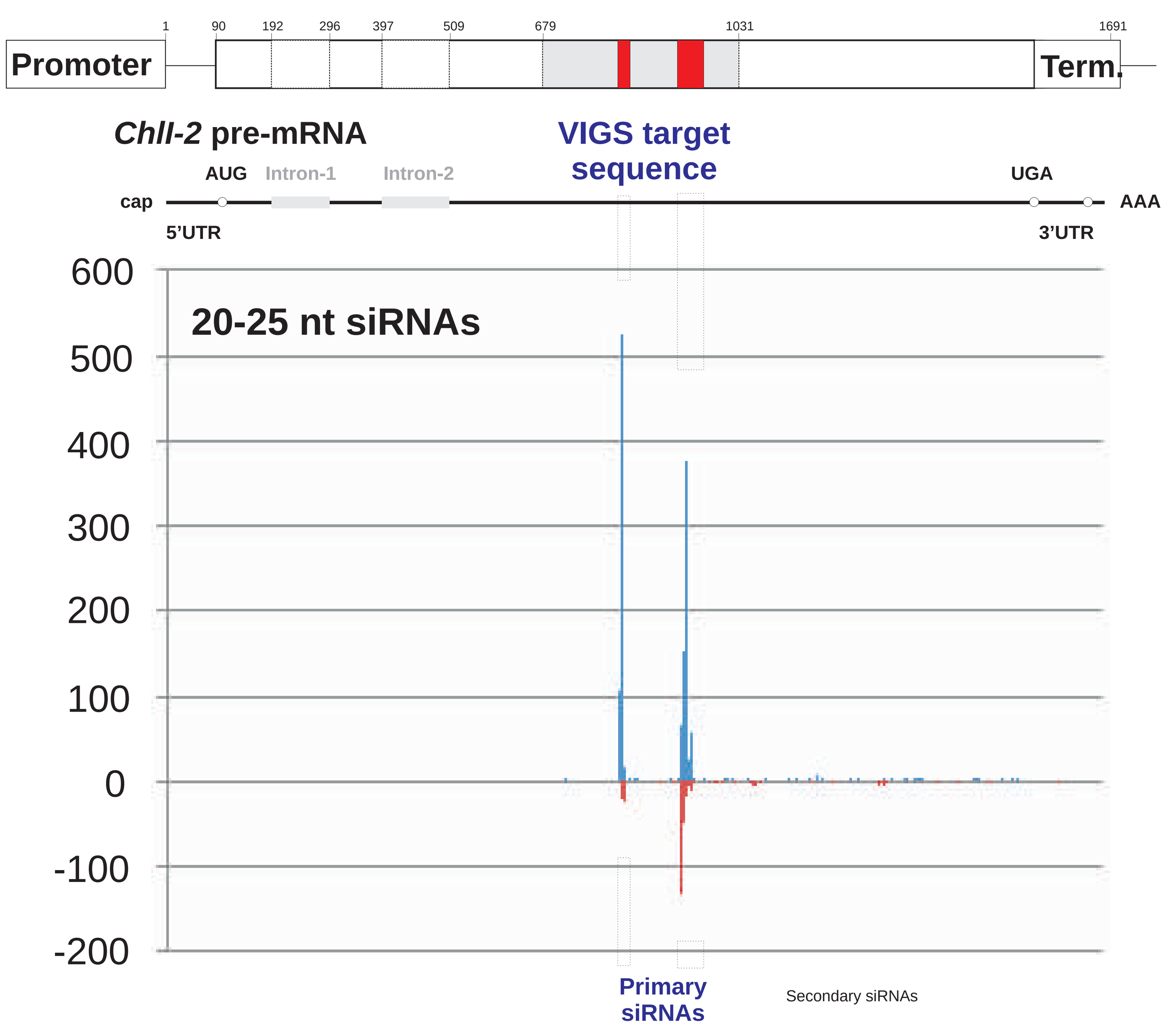

B.

Alignment of the VIGS target sequence (in blue) of *ChlI/CH42* and the homologous sequence of *ChlI-2* (two identical stretches of >20 nts are in red)

```
CH42     1176 TACAACTGTTAGATCTTTAGTTGATCTGTTACCTGAGATTAATGTAGTTG  1225
              .||||||||||||||||||||||||.||.|.||||||||.|.|||.|||.
ChlI2     662 AACAACTGTTAGATCTTTAGTTGATTTGCTTCCTGAGATCACTGTTGTTT  711

CH42     1226 CAGGTGACCCGTATAACTCGGATCCGATAGATCCTGAGTTTATGGGTGTT  1275
              |.|||||.|||||||||||.||.||||.|||||||||||.||||||....
ChlI2     712 CTGGTGATCCGTATAACTCAGACCCGAGAGATCCTGAGTGTATGGGAAAG  761

CH42     1276 GAAGTAAGAGAGAGAGTTGAGAAAGGAGAGCAAGTTCCTGTTATTGCGAC  1325
              |||||||||||||.||||.|.||.|||||..||.|..|||||||||..||
ChlI2     762 GAAGTAAGAGAGAAAGTTCAAAAGGGAGAAGAATTGTCTGTTATTGAAAC  811

CH42     1326 TAAGATTAATATGGTTGATCTTCCTTTGGGTGCAACAGAAGATAGAGTTT  1375
              .||.||.||||||||||||||||||||||||||.||.|||||||||||||
ChlI2     812 CAAAATCAATATGGTTGATCTTCCTTTGGGTGCTACTGAAGATAGAGTTT  861

CH42     1376 GTGGAACCATCGATATCGAAAAGGCTTTGACAGAAGGTGTAAAAGCCTTT  1425
              |||||||.||.|||||||||||||||||.||.||||||||.||.||.|||
ChlI2     862 GTGGAACTATTGATATCGAAAAGGCTTTAACCGAAGGTGTTAAGGCGTTT  911

CH42     1426 GAGCCTGGTTTGTTGGCTAAAGCTAATAGAGGGATTCTTTATGTTGATGA  1475
              ||||||||..|..|.|||||||||||||||||||||||||||||||||||
ChlI2     912 GAGCCTGGACTACTAGCTAAAGCTAATAGAGGGATTCTTTATGTTGATGA  961

CH42     1476 AGTTAATCTCTTGGATGATCATTTGGTTGATGTTCTTTTGGATTCAGCTG  1525
              |||||||||.||||||||||||||.||||||||||||.|.||||||||||
ChlI2     962 AGTTAATCTTTTGGATGATCATTTAGTTGATGTTCTTCTTGATTCAGCTG  1011

CH42     1526 CTTCTGGTTGGAATACGGTTGAGAGAGAAGGGATTTCGATTTCTCACCCG  1575
              |.|||||||||||.||.|||||.|||||||||||.|||||||||||.||.
ChlI2    1012 CATCTGGTTGGAACACTGTTGAAAGAGAAGGGATATCGATTTCTCATCCT  1061
```

C. Viral siRNAs

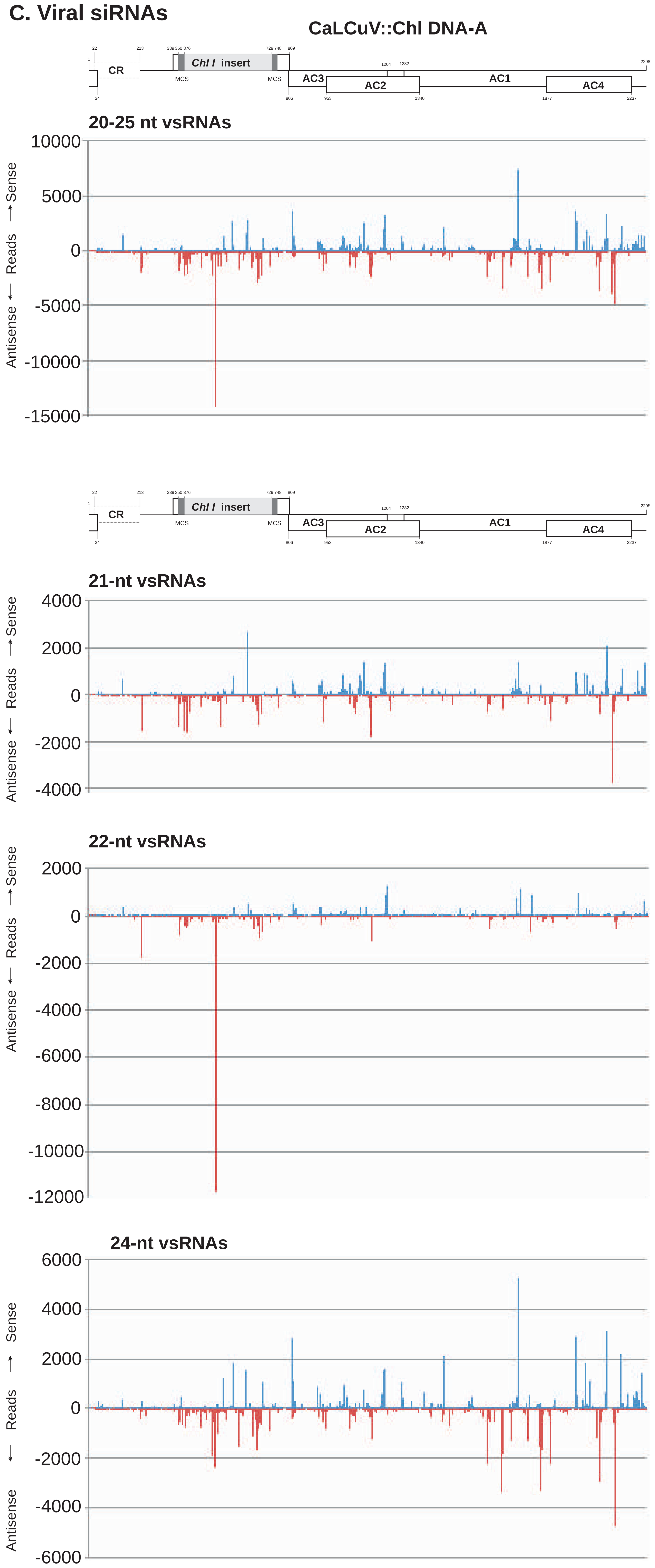

Figure S3. Viral and target gene siRNAs in CaLCuV::ChI virus-infected wild type (Col-0) plants. (A) The 1961 bp *ChlI-2* genomic locus is shown schematically; numbering starts from the transcription start site. The VIGS target region is highlighted in grey, with the two stretches of >20 nts in length which are identical in *ChlI* and *ChlI-2* shown in red. The graph plots the number of 20-25 nt siRNA reads at each nucleotide position of the *ChlI-2* gene; Bars above the axis represent sense reads starting at each respective position; those below represent antisense reads ending at the respective position (Table S4). **(B)** Alignment of the *ChlI* and *ChlI-2* sequences containing the VIGS target region is shown below the graph; **(C)** Virus-derived siRNAs. The graphs plot the number of 20-25 nt, 21-nt, 22-nt, or 24-nt vsRNA reads at each nucleotide position of the 2298 bp CaLCuV::ChI DNA-A. Bars above the axis represent sense reads starting at each respective position; those below represent antisense reads ending at the respective position (Tables S4). The genome organization of CaLCuV::ChI DNA is shown schematically above the graphs, with leftward (AC1, AC4, AC2, AC3 and BC1) ORFs and the rightward AV1::ChI chimeric gene and the common region (CR) indicated. The 353 bp *ChlI* gene segment inserted in the multiple cloning site (MCS) of the CaLCuV VIGS vector is highlighted in grey.
