# Supplementary material for: Primary and Secondary siRNAs in Geminivirus-induced Gene Silencing
Source: PLoS Pathog. 2012 Sep 27;8(9):e1002941. doi: 10.1371/journal.ppat.1002941 (PMC3460622; doi:10.1371/journal.ppat.1002941)
Supplement: Figure S4 — VIGS phenotypes and accumulation of primary and secondary siRNAs in L2 GFP transgenic plants infected with CaLCuV::GFP viruses. (A) The L2 T-DNA region containing the 35S-GFP transgene is shown schematically. Positions of the duplicated CaMV 35S enhancer and core promoter elements, GFP mRNA elements including 5′UTR, translation start (AUG) and stop (UAA) codons and 3′UTR with poly(A) signal (AAUAAA), and 35S terminator sequences are indicated. Numbering is from the T-DNA left border (LB). The VIGS target sequences, inserted in the CaLCuV::GFP viruses EnhSh, CodM, CodE and CodFL are indicated with dotted boxes. (B) Pictures under UV light of the L2 transgenic plant infected with the CodFL virus at 7, 12, 19, 26 and 33 days post-inoculation (dpi) and of the same plant at 40 dpi under UV and day light. Below are pictures under UV light of L2 plants infected with the CaLCuV empty vector and its derivatives EnhSh, CodM and CodE. Sampling of infected tissues of lower leaves (LL) and upper leaves (UL) for RNA preparation was performed as indicated on the left image. (C) Blot hybridization analysis of total RNA isolated from plants shown in Panel B. The blot was successively hybridized with short DNA probes specific for 35S::GFP transgene sequences inserted in the CaLCuV::GFP viruses EnhSh, CodM and CodE and for the GFP mRNA 3′UTR non-target sequence (3′UTR). EtBr staining serves as loading control. (D) Real time quantitative RT-PCR (qPCR) analysis of GFP mRNA accumulation in upper leaves of L2 plants infected with infected with the CaLCuV empty vector and its derivatives EnhSh, CodM, CodE (shown in Panel B). Total RNA from non-transgenic wild type Arabidopsis (Col-0) was used as a negative control. (PDF) [file ppat.1002941.s004.pdf]

Figure S4. VIGS phenotypes and accumulation of primary and secondary siRNAs in L2 GFP transgenic plants infected with CaLCuV::GFP viruses

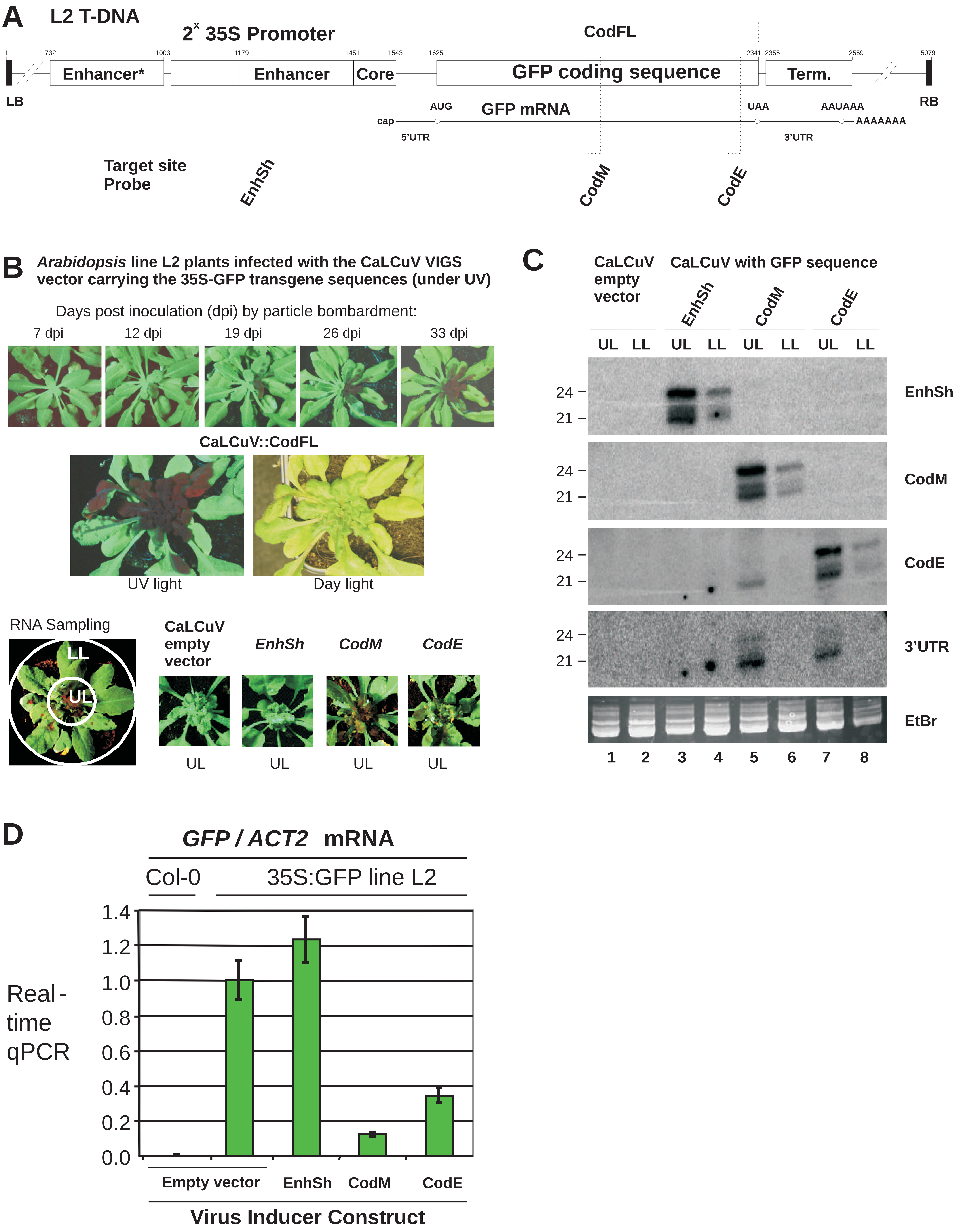

Figure S4. VIGS phenotypes and accumulation of primary and secondary siRNAs in L2 GFP transgenic plants infected with CaLCuV::GFP viruses. (A) The L2 T-DNA region containing the 35S-GFP transgene is shown schematically. Positions of the duplicated CaMV 35S enhancer and core promoter elements, GFP mRNA elements including 5'UTR, translation start (AUG) and stop (UAA) codons and 3'UTR with poly(A) signal (AAUAAA), and 35S terminator sequences are indicated. Numbering is from the T-DNA left border (LB). The VIGS target sequences, inserted in the CaLCuV::GFP viruses *EnhSh, CodM, CodE* and *CodFL* are indicated with dotted boxes. **(B)** Pictures under UV light of the L2 transgenic plant infected with the *CodFL* virus at 7, 12, 19, 26 and 33 days post-inoculation (dpi) and of the same plant at 40 dpi under UV and day light. Below are pictures under UV light of L2 plants infected with the CaLCuV empty vector and its derivatives *EnhSh, CodM* and *CodE*. Sampling of infected tissues of lower leaves (LL) and upper leaves (UL) for RNA preparation was performed as indicated on the left image. **(C)** Blot hybridization analysis of total RNA isolated from plants shown in Panel B. The blot was successively hybridized with short DNA probes specific for 35S::GFP transgene sequences inserted in the CaLCuV::GFP viruses *EnhSh, CodM* and *CodE* and for the GFP mRNA 3'UTR non-target sequence (3'UTR). EtBr staining serves as loading control. **(D)** Real time quantitative RT-PCR (qPCR) analysis of GFP mRNA accumulation in upper leaves of L2 plants infected with infected with the CaLCuV empty vector and its derivatives *EnhSh, CodM, CodE* (shown in Panel B). Total RNA from non-transgenic wild type *Arabidopsis* (Col-0) was used as a negative control.
