# Supplementary material for: Primary and Secondary siRNAs in Geminivirus-induced Gene Silencing
Source: PLoS Pathog. 2012 Sep 27;8(9):e1002941. doi: 10.1371/journal.ppat.1002941 (PMC3460622; doi:10.1371/journal.ppat.1002941)
Supplement: Figure S5 — Maps of primary and secondary siRNAs accumulating in L2 transgenic plants infected with CaLCuV::GFP viruses that target the GFP transcribed region. The graphs plot the number of 21-nt, 22-nt and 24-nt vsRNA reads at each nucleotide position of the L2 T-DNA-based 35S::GFP transgene in L2 transgenic plants infected with the CaLCuV::GFP viruses Lead (A), CodM (B), Trail (C), or PolyA (D). Bars above the axis represent sense reads starting at each respective position; those below represent antisense reads ending at the respective position (Table S5). The 35S-GFP transgene is shown schematically above the graphs. Positions of the duplicated 35S enhancer and core promoter, GFP mRNA elements and 35S terminator are indicated. Numbering is from the T-DNA left border (LB). The VIGS target sequences inserted in the CaLCuV::GFP viruses Lead, CodM, Trail or polyA are indicated with dotted boxes. (PDF) [file ppat.1002941.s005.pdf]

Figure S5. Maps of primary and secondary siRNAs accumulating in L2 transgenic plants infected with CaLCuV::GFP viruses that target the GFP transcribed region

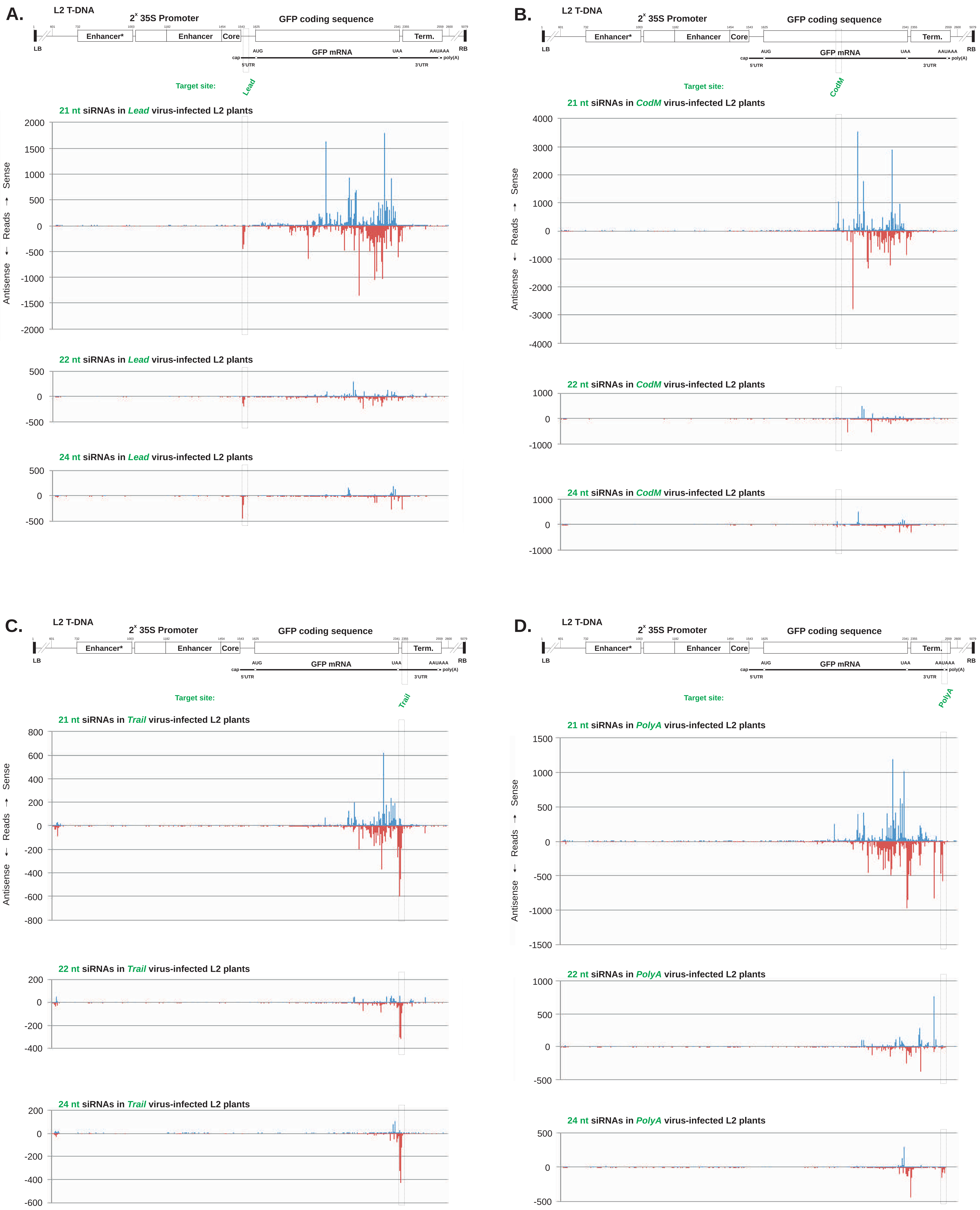

Figure S5. Maps of primary and secondary siRNAs accumulating in L2 transgenic plants infected with CaLCuV::GFP viruses that target the GFP transcribed region. The graphs plot the number of 21-nt, 22-nt and 24-nt vsRNA reads at each nucleotide position of the L2 T-DNA-based 35S::GFP transgene in L2 transgenic plants infected with the CaLCuV::GFP viruses *Lead* **(A)**, *CodM* **(B)**, *Trail* **(C)**, or *PolyA* **(D)**. Bars above the axis represent sense reads starting at each respective position; those below represent antisense reads ending at the respective position (Table S5). The 35S-GFP transgene is shown schematically above the graphs. Positions of the duplicated 35S enhancer and core promoter, GFP mRNA elements and 35S terminator are indicated. Numbering is from the T-DNA left border (LB). The VIGStarget sequences inserted in the CaLCuV::GFP viruses *Lead, CodM, Trail* or *polyA* are indicated with dotted boxes.
