# Supplementary material for: Primary and Secondary siRNAs in Geminivirus-induced Gene Silencing
Source: PLoS Pathog. 2012 Sep 27;8(9):e1002941. doi: 10.1371/journal.ppat.1002941 (PMC3460622; doi:10.1371/journal.ppat.1002941)
Supplement: Figure S6 — Maps of primary siRNAs accumulating in L2 transgenic plants infected with CaLCuV::GFP viruses that target the GFP promoter elements. The graphs plot the number of 21-nt, 22-nt and 24-nt vsRNA reads at each nucleotide position of the L2 T-DNA-based 35S::GFP transgene in L2 transgenic plants infected with the CaLCuV::GFP viruses Enh (A), ProFL (B) or Core (C). Bars above the axis represent sense reads starting at each respective position; those below represent antisense reads ending at the respective position (Table S5). The 35S-GFP transgene is shown schematically above the graphs. Positions of the duplicated 35S enhancer and core promoter, GFP mRNA elements and 35S terminator are indicated. Numbering is from the T-DNA left border (LB). The VIGS target sequences inserted in the CaLCuV::GFP viruses Enh, CodFL and Core are indicated with dotted boxes. Note that the duplicated 35S promoter sequences Enhancer* and Enhancer (each 273 nt long) share 94% nucleotide identity, since they originate from two different strains of CaMV. Therefore, primary siRNA reads are unequally distributed between the two VIGS target regions. (PDF) [file ppat.1002941.s006.pdf]

Figure S6. Maps of primary siRNAs accumulating in L2 transgenic plants infected with CaLCuV::GFP viruses that target the GFP promoter elements.

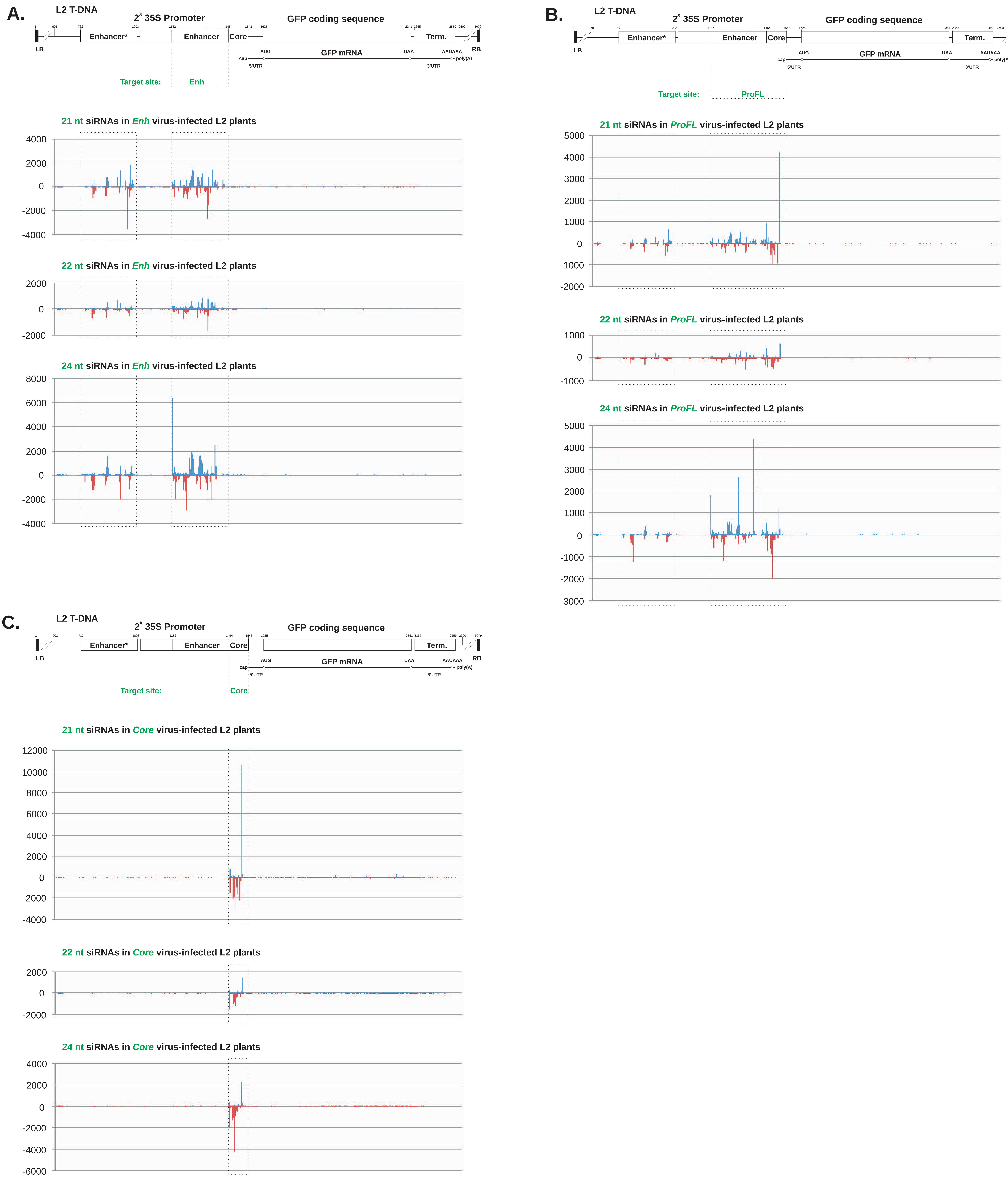

Figure S6. Maps of primary siRNAs accumulating in L2 transgenic plants infected with CaLCuV::GFP viruses that target the GFP promoter elements. The graphs plot the number of 21-nt, 22-nt and 24-nt vsRNA reads at each nucleotide position of the L2 T-DNA-based 35S::GFP transgene in L2 transgenic plants infected with the CaLCuV::GFP viruses *Enh* **(A)**, *ProFL* **(B)** or *Core* **(C)**. Bars above the axis represent sense reads starting at each respective position; those below represent antisense reads ending at the respective position (Table S5). The 35S-GFP transgene is shown schematically above the graphs. Positions of the duplicated 35S enhancer and core promoter, GFP mRNA elements and 35S terminator are indicated. Numbering is from the T-DNA left border (LB). The VIGS target sequences inserted in the CaLCuV::GFP viruses *Enh, CodFL* and *Core* are indicated with dotted boxes. Note that the duplicated 35S promoter sequences Enhancer* and Enhancer (each 273 nt long) share 94% nucleotide identity, since they originate from two different strains of CaMV. Therefore, primary siRNA reads are unequally distributed between the two VIGS target regions.
