# Supplementary material for: Primary and Secondary siRNAs in Geminivirus-induced Gene Silencing
Source: PLoS Pathog. 2012 Sep 27;8(9):e1002941. doi: 10.1371/journal.ppat.1002941 (PMC3460622; doi:10.1371/journal.ppat.1002941)
Supplement: Protocol S1 — The file contains the list of DNA oligonucleotides probes for RNA and DNA blot hybridization, primers for subcloning of the 35S::GFP tarnsgene-derived sequences into CaLCuV VIGS vector and for real time PCR as well as Reference sequences used for bioinformatic analysis. (PDF) [file ppat.1002941.s007.pdf]

Supplementary Protocol S1

DNA oligonucleotides used as probes for RNA and DNA blot hybridization, for subcloning of the 35S::GFP tarnsgene-derived sequences into CaLCuV VIGS vector and real time PCR

Name original	Detects	Gene/Region	Sequence
Arabidopsis genome specific probes:			
siR255_as	siR255	TAS1a/b/c	5’-TACGCTATGTTGGACTTAGAA
miR173_as	miR173	miRNA	5’-GTGATTTCTCTCTGCAAGCGAA
siR1003 as	siR1003	ra-siRNA	5’-ATGCCTATGTTGGCCTCACGGTCT
Met_tRNA_as	Met-tRNA	tRNA	5’-TGGTATCAGAGCCAGGTTTCGATCC
CaLCuV-specific probes DNA-A:			
CbA211_s	vsRNA	IGR	5’-gtgggaccacgcattaaatgaaatctaacc
CbA211_as	vsRNA	IGR	5’-ggttagatttcatttaatgcgtggtcccac
CbA455_s	vsRNA	AV1	5’-ggttaacaggcccatgtacaggaagcccag
CbA455_as	vsRNA	AV1	5’-ctgggcttcctgtacatgggcctgttaacc
CbA675_s	vsRNA	AV1	5’-ggcaagatatggatggacgagaatatcaag
CbA675_as	vsRNA	AV1	5’-cttgatattctcgtccatccatatcttgcc
CbA1063_s	vsRNA	AV1/Term	5’-CGGATCTATTTTTACGATTCGATAAC
CbA1063_as	vsRNA	AV1/Term	5’-GTTATCGAATCGTAAAAATAGATCCG
CbA1113_s	vsRNA	AC3/Term	5’-acatgattctcgtgaacatgagttacataa
CbA1113_as	vsRNA	AC3/Term	5’-ttatgtaactcatgttcacgagaatcatgt
CbA1484_s	vsRNA	AC2	5’-atccattatctgcgcaattgatgtggaggaa
CbA1484_as	vsRNA	AC2	5’-ttcctccacatcaattgcgcagataatggat
CbA1652_s	vsRNA	AC1/AC2-Promoter	5’-gcattttcctctttgttgaggaaacttat
CbA1652_as	vsRNA	AC1/AC2-Promoter	5’-ataagtttcctcaacaaagaggaaaatgc
CbA1874_s	vsRNA	AC1	5’-tttgaattaaagtcgaggtgcccactcaaa
CbA1874_as	vsRNA	AC1	5’-tttgagtgggcacctcgactttaattcaaa
CbA2186_s	vsRNA	AC4	5’-gcctcttccacacatcccgcattgacggcg
CbA2186_as	vsRNA	AC4	5’-cgccgtcaatgcgggatgtgtggaagaggc
AC4_s/CbA2295_s	vsRNA	AC4	5’-TGGTGATGTAATTCTTGACGGCATTGGTGTCT
AC4_as/CbA2295_as	vsRNA	AC4	5’-AGACACCAATGCCGTCAAGAATTACATCACCA
CaLCuV common region (CR)-specific probes DNA-A and DNA-B:			
CbB23_s	vsRNA	CR	5’-gttcatggacaccaggagagctctcgtcta
CbB23_as	vsRNA	CR	5’-tagacgagagctctcctggtgtccatgaac
CbB159_s	vsRNA	CR	5’-ggatggccgcaaatttttggagtcctggtg
CbB159_as	vsRNA	CR	5’-caccaggactccaaaaatttgcggccatcc
CaLCuV-specific probes DNA-B:			
CbB500_s	vsRNA	BV1 5’UTR	5’-caaatcattttgtagaggtgctgagaataa
CbB500_as	vsRNA	BV1 5’UTR	5’-ttattctcagcacctctacaaaatgatttg
CbB521_s	vsRNA	BV1	5’-tgagaataatgtatcctacaaagtttaggc
CbB521_as	vsRNA	BV1	5’-gcctaaactttgtaggatacattattctca
CbB611_s	vsRNA	BV1	5’-cttttgttagacgcactgatgggaaacgtc
CbB611_as	vsRNA	BV1	5’-gacgtttcccatcagtgcgtctaacaaaag
CbB1321_s	vsRNA	IGR	5’-gattgaatatatgcattaataataaaacac
CbB1321_as	vsRNA	IGR	5’-gtgttttattattaatgcatatattcaatc

CbB1621_s	vsRNA	BC1	5′-atcacctggttgtaattcaattgggcctgg
CbB1621_as	vsRNA	BC1	5′-ccaggcccaattgaattacaaccaggtgat
CbB1697_s	vsRNA	BC1	5′-ctctcccatcttccgtagtccacatgtgag
CbB1697_as	vsRNA	BC1	5′-ctcacatgtggactacggaagatgggagag
CbB1755_s	vsRNA	BC1	5′-ttgaatgaattttcactgttggtgcccgga
CbB1755_as	vsRNA	BC1	5′-tccgggcaccaacagtgaaaattcattcaa
CbB1891_s	vsRNA	BC1	5′-tttccacggaatggggtcttttagagagaa
CbB1891_as	vsRNA	BC1	5′-ttctctctaaaagaccccattccgtggaaa
CbB2237_s	vsRNA	BC1 5′UTR	5′-gaattcatttatagaaatccgaccgcgcag
CbB2237_as	vsRNA	BC1 5′UTR	5′-ctgcgcggtcggatttctataaatgaattc
CbB2459_s	vsRNA	BC1 Promoter	5′-tctgattatatagagagcaaatttgaagaa
CbB2459_as	vsRNA	BC1 Promoter	5′-ttcttcaaatttgctctctatataatcaga

Chl insert-specific probes:

*Chl_all*_s	Chl_s	ChlorataI/CH42	5′-AATATGGTTGATCTTCCTTTGGGTGCAACAG
*Chl_all*_as	Chl_as	Chlorata I/CH42	5′-CTGTTGCACCCAAAGGAAGATCAACCATATT

L2 transgene-specific probes for blot hybridization:

L2lead_s	Lead_s	L2 GFP 5′UTR	5′-CTTCAACAATTACCAACAACAACAAACAAC
L2start_s	Start_s	L2 GFP ATG	5′-ATTTACAATTACACCATGGGTAAGGGAGAG
L2code_s	CodB_s	L2 GFP Coding	5′-GAGCTCTTCACCGGGGTGGTGCCCATCCTG
L2midGFP_s	CodM_s	L2 GFP Coding	5′-CGCATCGAGCTGAAGGGCATCGACTTCAAG
L2endGFP_s	CodE_s	L2 GFP Coding	5′-GCGATCACATGGTCCTGCTGGAGTTCGTGA
L2stop_s	Stop_s	L2 GFP TAA	5′-TGAACTATACAAATAAAGGATCCTCTAGAG
L2term_s	Trail_s	L2 GFP 3′UTR	5′-TCCGCAAAAATCACCAGTCTCTCTCTACAA
L2_TERM_as	Trail_as	L2 GFP 3′UTR	5′-tcgagTTGTAGAGAGAGACTGGTGATTTTTGCGGAt
L2_POLYA_s	PolyA_s	L2 GFP 3′UTR	5′-ctagaTTCTATCAATAAAATTTCTAATTCCTAAAAc
L2_3UTR_s	3′UTR	L2 GFP 3′UTR	5′-GTGTGAGTAGTTCCCAGATAAGGGAATTAGGGT-
			-TCTTATAGGGTTTCG
L2caat_s	CAAT_s	L2 35S Core	5′-GGATGACGCACAATCCCACTATCCTTCGCA
L2tata_s	TATA_s	L2 35S Core	5′-AGACCCTTCCTCTATATAAGGAAGTTCATT
L2plus1_s	Plus1_s	L2 35S Core	5′-TCATTTGGAGAGGACAGGCTTCTTGAGATC
L2codbeg_s	Codbeg_s	L2 GFP Coding	5′-GTCGAGCTGGACGGCGACGTAAACGGC-
			-CACAAGTTCAGCGTGTCCGGC
L2codmid_s	Codmid_s	L2 GFP Coding	5′-GGGCATCGACTTCAAGGAGGACGGCAA-
			-CATCCTGGGGCACAAGCTGGA
L2codend_s	Codend_s	L2 GFP Coding	5′-GGTCCTGCTGGAGTTCGTGACCGCCGC-
			-CGGGATCACTCACGGCATGGA
L2codend_as	Codend_as	L2 GFP Coding	5′-TCCATGCCGTGAGTGATCCCGGCGGCG-
			-GTCACGAACTCCAGCAGGACC
L2PostProcess_s	Post_s	L2 post polyA	5′-AGTGACCTGCAGGCATGCAAGCTTGGCGTA
L2PostProcess_as	Post	L2 post polyA	5′-TACGCCAAGCTTGCATGCCTGCAGGTCACT
L2_35Senh_s1	Enh_s1	L2 35S Enhancer	5′-GTCCCAAAGATGGACCCCCACCCACGAG-
			-GAGCATCGTGGAAAA
L2_35Senh_s2	Enh_s2	L2 35S Enhancer	5′-ACGAGGAGCATCGTGGAAAAAGAAGACG-
			-TTCCAACCACTGTCTT
L2_35Senh_s3	Enh_s3	L2 35S Enhancer	5′-GACGTTCCAACCACTGTCTTCAAAGCAA-
			-GTGGATTGATGTGAT
L2_35Senh_as1	Enh_as1	L2 35S Enhancer	5′-ATCACATCAATCCACTTGCTTTGAAGACA-
			-GTGGTTGGAACGTC
L2_35Senh_as2	Enh_as2	L2 35S Enhancer	5′-AAGACAGTGGTTGGAACGTCTTCTTTTTC-
			-CACGATGCTCCTCGT
L2_35Senh_as3	Enh_as3	L2 35S Enhancer	5′-TTTTCCACGATGCTCCTCGTGGGTGGGGG-
			-TCCATCTTTGGGAC
L2_35Score_s1	Core_s1	L2 35S Core	5′-ATCTCCACTGACGTAAGGGATGACGCACA-
			-ATCCCACTATCCTT
L2_35Score_s2	Core_s2	L2 35S Core	5′-CGCACAATCCCACTATCCTTCGCAAGACC-
			-CTTCCTCTATATAAG
L2_35Score_s3	Core_s3	L2 35S Core	5′-AGACCCTTCCTCTATATAAGGAAGTTCAT-
			-TTCATTTGGAGAGG
L2_35Score_as1	Core_as1	L2 35S Core	5′-CCTCTCCAAATGAAATGAACTTCCTTATAT-
			-AGAGGAAGGGTCT

L2_35Score_as2	Core_as2	L2 35S Core	5’-CTTATATAGAGGAAGGGTCTTGCGAAGGAT-<br>-AGTGGGATTGTGCG
L2_35Score_as3	Core_as3	L2 35S Core	5’-AAGGATAGTGGGATTGTGCGTCATCCCTTA-<br>-CGTCAGTGGAGAT

<u>L2 short insert cloning oligos (XbaI-XhoI):</u>

L2_35Sfrag_s	EnhSh	L2 35S Enhancer	5’-CTAGAGGGTAATATCCGGAAACCTCCTCGGATTCC
L2_35Sfrag_as	EnhSh	L2 35S Enhancer	5’-TCGAGGAATCCGAGGAGGTTTCCGGATATTACCCT
L2_CAAT_s	CAAT	L2 35S Core	5’-ctagaGGATGACGCACAATCCCACTATCCTTCGCAc
L2_CAAT_as	CAAT	L2 35S Core	5’-tcgagTGCGAAGGATAGTGGGATTGTGCGTCATCCt
L2_TATA_s	TATA	L2 35S Core	5’-ctagaAGACCCTTCCTCTATATAAGGAAGTTCATTc
L2_TATA_as	TATA	L2 35S Core	5’-tcgagAATGAACTTCCTTATATAGAGGAAGGGTCTt
L2_PLUS1_s	Plus1	L2 35S Core	5’-ctagaTCATTTGGAGAGGACAGGCTTCTTGAGATCc
L2_PLUS1_as	Plus1	L2 35S Core	5’-tcgagGATCTCAAGAAGCCTGTCCTCTCCAAATGAt
L2_LEAD_s	Lead	L2 GFP 5’UTR	5’-ctagaCTTCAACAATTACCAACAACAACAAACAACc
L2_LEAD_as	Lead	L2 GFP 5’UTR	5’-tcgagGTTGTTTGTTGTTGTTGGTAATTGTTGAAGt
L2_START_s	Start	L2 GFP ATG	5’-ctagaATTTACAATTACACCATGGGTAAGGGAGAGc
L2_START_as	Start	L2 GFP ATG	5’-tcgagCTCTCCCTTACCCATGGTGTAATTGTAAATt
L2_CODE_s	CodB	L2 GFP Coding	5’-ctagaGAGCTCTTCACCGGGGTGGTGCCCATCCTGc
L2_CODE_as	CodB	L2 GFP Coding	5’-tcgagCAGGATGGGCACCACCCCGGTGAAGAGCTCt
L2_midGFPfrag_s	CodM	L2 GFP Coding	5’-CTAGACGCATCGAGCTGAAGGGCATCGACTTCAAGC
L2_midGFPfrag_as	CodM	L2 GFP Coding	5’-TCGAGCTTGAAGTCGATGCCCTTCAGCTCGATGCGT
L2_3’GFPfrag_s	CodE	L2 GFP Coding	5’-CTAGAGCGATCACATGGTCCTGCTGGAGTTCGTGAC
L2_3’GFPfrag_as	CodE	L2 GFP Coding	5’-TCGAGTCACGAACTCCAGCAGGACCATGTGATCGCT
L2_STOP_s	Stop	L2 GFP TAA	5’-ctagaTGAACTATACAAATAAAGGATCCTCTAGAGc
L2_STOP_as	Stop	L2 GFP TAA	5’-tcgagCTCTAGAGGATCCTTTATTTGTATAGTTCAt
L2_TERM_s	Trail	L2 GFP 3’UTR	5’-ctagaTCCGCAAAAATCACCAGTCTCTCTCTACAAc
L2_TERM_as	Trail	L2 GFP 3’UTR	5’-tcgagTTGTAGAGAGAGACTGGTGATTTTTGCGGAt
L2_POLYA_s	PolyA	L2 GFP 3’UTR	5’-ctagaTTCTATCAATAAAATTTCTAATTCCTAAAAc
L2_POLYA_as	PolyA	L2 GFP 3’UTR	5’-tcgagTTTTAGGAATTAGAAATTTTATTGATAGAAt
L2_POST_s	Post	L2 post polyA	5’-ctagaAGTGACCTGCAGGCATGCAAGCTTGGCGTAc
L2_POST_as	Post	L2 post polyA	5’-tcgagTACGCCAAGCTTGCATGCCTGCAGGTCACTt

<u>L2 PCR primers for cloning 35S Enhancer, Core and ProFL as XbaI-XhoI fragments:</u>

L2Enh_Xba_s	Enh	L2 35S Enhancer	5’-CAGTtctAGAAGACCAAAGGGCAATTGAGAC
L2Enh_Xho_as	Enh	L2 35S Enhancer	5’-CAGTctcGAgATCACATCAATCCACTTGCTTTG
L2Pro_Xba_s	Core	L2 35S Core	5’-TGATtctAgATCTCCACTGACGTAAGGGATGACGC
L2Pro_Xho_as	Core	L2 35S Core	5’-AGAActCgagCCTCTCCAAATGAAATGAACTTCC
L2Enh_Xba_s	ProFL	L2 35S Enhancer	5’-CAGTtctAGAAGACCAAAGGGCAATTGAGAC
L2Pro_Xho_as	ProFL	L2 35S Core	5’-AGAActCgagCCTCTCCAAATGAAATGAACTTCC

<u>L2 PCR primers for cloning the complete GFP coding sequence CodFL:</u>

CaLCuV-AV2_GFP_F1	CodFL	GFP coding seq	5’-AGCGGTACCTATGGGTAAGGGAGAGGAGCT-<br>-CTTCACC
CaLCuV-AV1_GFP_R1	CodFL	GFP coding seq	5’-ATAGGTACCTTATTTGTATAGTTCATCCATGC-<br>-CGTGAGTG

<u>Primers for (RT)-qPCR:</u>

CbAV1_qPCR_s	AV1 mRNA	AV1	5’-ACAGGAAGCCCAGGATTTATAGGAC
CbAV1_qPCR_as			5’-AAATGTCATGCCGCTGCTCATAAG
CbAC1_qPCR_s	AC1/4 mRNA	AC1	5’-ACCCGTCTGAATGTTCCTCTCTTG
CbAC1_qPCR_as			5’-ACAGTGCGACATACCCAAAGATGAAG

CbAC3_qPCR_s	AC2/3 + AC1/4	AC3	5’-AAGCTCTCATCGAAGTCGTCCAG
CbAC3_qPCR_as	mRNAs		5’-ACCAGAACCAGGATCTACCACATC
CbBV1_qPCR_s	BV1 mRNA	BV1	5’-GACCCATAACTCAGCCCTTTCAAC
CbBV1_qPCR_as			5’-CACGCTCAATCTTAACGGTTCCC
CbBC1_qPCR_s	BC1 mRNA	BC1	5’-CCCTTTGTAACCCAGCTTGTGATG
CbBC1_qPCR_as			5’-TCCCAACCCACACACAGAATCAG
L2GFP_qPCR_s	GFP mRNA	L2 GFP	5'-ACCATCTTCTTCAAGGACGACGG
L2GFP_qPCR_as			5'-GTTGTGGCTGTTGTAGTTGTACTCC

<u>Actin 2 (internal control for qRT-PCT)</u> – 206 bp for spliced RNA; 283 bp for genomic DNA

ACT2_qPCR2_s	ACT2 mRNA	ACT2	5'-GCACCCTGTTCTTCTTACCG-3'
ACT2_qPCR2_as			5'-AACCCTCGTAGATTGGCACA-3'

<u>18S rDNA (internal control for qPCR)</u>

18S_qPCRshort_s	18S rDNA	18S rDNA	5’-GGTGGTAACGGGTGACG-3’
18S_qPCRshort_as			5’-CGCCTGCTGCCTTCC-3’

<u>Probes for detection of CaLCuV long RNAs:</u>

AV1 as mix = CbA455_as + CbA675_as + CbAV1_qPCR_as
AC2 s mix = CbA1113_s + CbA1484_s + CbAC3_qPCR_s
BV1 as mix = CbB985_as + CbB521_as + CbB611_as + CbBV1_qPCR_as
BC1 s mix = CbB1621_s + CbB1697_s + CbB1755_s + CbB1891_s + CbBC1_qPCR_s

Reference sequences used for bioinformatic analysis

CaLCuV DNA-A

>CaLCuV-A

[illegible]

CaLCuV DNA-B

>CaLCuV-B

[illegible]

CaLCuV-ChI

>CbChl

[illegible]

Chlorata I/*ChlI-1*/*CH42* genomic locus

>CH42

[illegible]

CH42 insert/target

>CH42_insert

[illegible]

ChlI-2 genomic locus

>CHLI-2

[illegible]

CaLCuV-Lead

>CbL2_Lead

[illegible]

CaLCuV-CodM

>CbL2_midGFP

[illegible]

CaLCuV-Trail

>CbL2_Term

[illegible]

ACCCTCGAGTTGTAGAGAGAGACTGGTGATTTTTGCGGATCTAGAGGATCCAGATCTGAGCTCGCTAGCCTAATCCTGTGTCTGCGACATTAAAAATTCGGATCTATTTTTACGATTCGATAACAAATTAATAAATTTTGAAATTTATTACATGATTCTCGTGAACATGAGTTACATAG
GATCTGTCCGTTGCGAAACGAACAGCTCTAATTACATGATTAATACCAATAACACCTAGGTTATCTAAATGAGACATTACAAGGCATTTGAATCTACTTAAATATGTCGGCCCAGAAGCTCTCATCGAAGTCGTCCAGACTTGGAAGTTGAAGTAGGCTTTGCGGAGATCCAATGCTTT
CCGTAGGTTGTGGTTGAACCGGACTTGGATGTGGTAGATCCTGGTTCTGGTGTACAACGGGTCCTCTACGCGTTGTATCCTGAAATATAGGGGATTTGGAACCTCCCAGATAAAAACGGAATTCTCTGCCTGAGCTACAGTGATGCTCTCCCCGGTGCGTGAATCCATTATCTGCGCAA
TTGATGTGGAGGAAGATAGAACACCCGCAGTTCAAATCAATGCGTCTTCGGCGTACAGCTCTCCTTTTAGCAATCTTGTGCTGTGCTTTGATAGAGGGGGGCTTCAAGAGTGATGAATTTTGCATTTTTGGTAGTCCACGCTCTTAGTGATGCATTTTCCTCTTTGTTGAGGAAACTTA
TATAACTGCTCCCCTCTCCTGGATTGCACAGCACGATTGAGGGTATGCCACCTTTAATTTGAACTGGCTTGCCGTACTTACAGTTTGATTGCCAGTCCCTTTGGGCCCCAATAAGCTCTTTCCAGTGCTTTAGCTTTAGATAATGCGGAGCTATGTCATCAATGACGTTATACTCCGCA
TTATTTGAAAAGACCTTTGAATTAAAGTCGAGGTGCCCACTCAAATAATTATGTGGTCCTAAAGCACGCGCCCACATGGTCTTGCCGGTTCGTGAATCACCTTCAACTATGATACTAATAGGTCTTTCCGCCCGCGCAGCGGCACTCCGACCAAAATAGTCATCTGCCCATGAACTCAT
CTCGTCCGGGACGTTAGTAAAGGAGGAGAGTTGAAACGGAGGAGCCCATGGTTCCGGAGCCTTAGTAAAGAGTCGCTCTATGTTAGCTCTAACATTATGATAACTAACAATGAACGTCTTTGGATCTCCAGCCCTTATAATTGCGAGAGCCTCTTCCACACATCCCGCATTGACGGCGT
TGTGGTAGACGTCGTCTTTATTTGCCTTTGTACCCCCAGACACCTTGTACTGCCCGGATTCACAATAATCACCATCTTTGGTGATGTAATTCTTGACGGCATTGGTGTCTTTGGCTGCCTGAATGTTTGGGTGAAAATTGGCAGACCTTCTGGGGTGAGTGATGTCGAAAAATCTAGCA
TCCTTGATGTTCGACTTTCCTGATAGTTGGATGAGACAGTGTAAATGGGGGAACCCGTCTGAATGTTCCTCTCTTGCGACTCTGATGTATGTGGGTTTGACGACTGACCACGACAGGGTTTGAAGCATCTGAAGAGCTTCATCTTTGGGTATGTCGCACTGGGGATATGTTAAGAATAT
ATTTCGGGC

CaLCuV-PolyA

>CbL2_polyA
TGCTAAACGAAACGATTTAGGGTTTCGTGGCATTTTTGTAAATAATATTTAGGACTCCAGGGGAGGACGCCAGGAGAGCTCTCTCTAAAACCTATTGTTTTTGGTGTCTTGGTGTCCAATATATACTAAAAGCCTCTGGGGAACACCAGGGGCAAAAGCGGCCATCCGCAATAATATTA
CCGGATGGCCGCGATTTTTTTGGTGTCTTGCGTGGGACCACGCATTAAATGAAATCTAACCAATCACATGCCGCCTGACAAGGTTAGCTATTAAGGCTAACTGAGTGCGCTGTGGGCCTATATAAAGACATGTGATTTCAATTTATGCTTTACTTCGAAAaGCCTAAGCGGCaAGCGGT
ACCCTCGAGTTTTAGGAATTAGAAATTTTATTGATAGAATCTAGAGGATCCAGATCTGAGCTCGCTAGCCTAATCCTGTGTCTGCGACATTAAAAATTCGGATCTATTTTTACGATTCGATAACAAATTAATAAATTTTGAAATTTATTACATGATTCTCGTGAACATGAGTTACATAG
GATCTGTCCGTTGCGAAACGAACAGCTCTAATTACATGATTAATACCAATAACACCTAGGTTATCTAAATGAGACATTACAAGGCATTTGAATCTACTTAAATATGTCGGCCCAGAAGCTCTCATCGAAGTCGTCCAGACTTGGAAGTTGAAGTAGGCTTTGCGGAGATCCAATGCTTT
CCGTAGGTTGTGGTTGAACCGGACTTGGATGTGGTAGATCCTGGTTCTGGTGTACAACGGGTCCTCTACGCGTTGTATCCTGAAATATAGGGGATTTGGAACCTCCCAGATAAAAACGGAATTCTCTGCCTGAGCTACAGTGATGCTCTCCCCGGTGCGTGAATCCATTATCTGCGCAA
TTGATGTGGAGGAAGATAGAACACCCGCAGTTCAAATCAATGCGTCTTCGGCGTACAGCTCTCCTTTTAGCAATCTTGTGCTGTGCTTTGATAGAGGGGGGCTTCAAGAGTGATGAATTTTGCATTTTTGGTAGTCCACGCTCTTAGTGATGCATTTTCCTCTTTGTTGAGGAAACTTA
TATAACTGCTCCCCTCTCCTGGATTGCACAGCACGATTGAGGGTATGCCACCTTTAATTTGAACTGGCTTGCCGTACTTACAGTTTGATTGCCAGTCCCTTTGGGCCCCAATAAGCTCTTTCCAGTGCTTTAGCTTTAGATAATGCGGAGCTATGTCATCAATGACGTTATACTCCGCA
TTATTTGAAAAGACCTTTGAATTAAAGTCGAGGTGCCCACTCAAATAATTATGTGGTCCTAAAGCACGCGCCCACATGGTCTTGCCGGTTCGTGAATCACCTTCAACTATGATACTAATAGGTCTTTCCGCCCGCGCAGCGGCACTCCGACCAAAATAGTCATCTGCCCATGAACTCAT
CTCGTCCGGGACGTTAGTAAAGGAGGAGAGTTGAAACGGAGGAGCCCATGGTTCCGGAGCCTTAGTAAAGAGTCGCTCTATGTTAGCTCTAACATTATGATAACTAACAATGAACGTCTTTGGATCTCCAGCCCTTATAATTGCGAGAGCCTCTTCCACACATCCCGCATTGACGGCGT
TGTGGTAGACGTCGTCTTTATTTGCCTTTGTACCCCCAGACACCTTGTACTGCCCGGATTCACAATAATCACCATCTTTGGTGATGTAATTCTTGACGGCATTGGTGTCTTTGGCTGCCTGAATGTTTGGGTGAAAATTGGCAGACCTTCTGGGGTGAGTGATGTCGAAAAATCTAGCA
TCCTTGATGTTCGACTTTCCTGATAGTTGGATGAGACAGTGTAAATGGGGGAACCCGTCTGAATGTTCCTCTCTTGCGACTCTGATGTATGTGGGTTTGACGACTGACCACGACAGGGTTTGAAGCATCTGAAGAGCTTCATCTTTGGGTATGTCGCACTGGGGATATGTTAAGAATAT
ATTTCGGGC

CaLCuV-Enh

>Cb35SEnh
TGCTAAACGAAACGATTTAGGGTTTCGTGGCATTTTTGTAAATAATATTTAGGACTCCAGGGGAGGACGCCAGGAGAGCTCTCTCTAAAACCTATTGTTTTTGGTGTCTTGGTGTCCAATATATACTAAAAGCCTCTGGGGAACACCAGGGGCAAAAGCGGCCATCCGCAATAATATTA
CCGGATGGCCGCGATTTTTTTGGTGTCTTGCGTGGGACCACGCATTAAATGAAATCTAACCAATCACATGCCGCCTGACAAGGTTAGCTATTAAGGCTAACTGAGTGCGCTGTGGGCCTATATAAAGACATGTGATTTCAATTTATGCTTTACTTCGAAAaGCCTAAGCGGCaAGCGGT
ACCCTCGAGTATCACATCAATCCACTTGCTTTGAAGACAGTGGTTGGAACGTCTTCTTTTTCCACGATGCTCCTCGTGGGTGGGGGTCCATCTTTGGGACCACTGTCGGCAGAGGCATCTTCAACGATGGCCTTTCCTTTATCGCAATGATGGCATTTGTAGGAGCCACCTTCCTTTTC
CACTATCTTCACAATAAAGTGACAGATAGCTGGGCAATGGAATCCGAGGAGGTTTCCGGATATTACCCTTTGTTGAAAAGTCTCAATTGCCCTTTGGTCTTCTAGAGGATCCAGATCTGAGCTCGCTAGCCTAATCCTGTGTCTGCGACATTAAAAATTCGGATCTATTTTTACGATTC
GATAACAAATTAATAAATTTTGAAATTTATTACATGATTCTCGTGAACATGAGTTACATAGGATCTGTCCGTTGCGAAACGAACAGCTCTAATTACATGATTAATACCAATAACACCTAGGTTATCTAAATGAGACATTACAAGGCATTTGAATCTACTTAAATATGTCGGCCCAGAAG
CTCTCATCGAAGTCGTCCAGACTTGGAAGTTGAAGTAGGCTTTGCGGAGATCCAATGCTTTCCGTAGGTTGTGGTTGAACCGGACTTGGATGTGGTAGATCCTGGTTCTGGTGTACAACGGGTCCTCTACGCGTTGTATCCTGAAATATAGGGGATTTGGAACCTCCCAGATAAAAACG
GAATTCTCTGCCTGAGCTACAGTGATGCTCTCCCCGGTGCGTGAATCCATTATCTGCGCAATTGATGTGGAGGAAGATAGAACACCCGCAGTTCAAATCAATGCGTCTTCGGCGTACAGCTCTCCTTTTAGCAATCTTGTGCTGTGCTTTGATAGAGGGGGGCTTCAAGAGTGATGAAT
TTTGCATTTTTGGTAGTCCACGCTCTTAGTGATGCATTTTCCTCTTTGTTGAGGAAACTTATATAACTGCTCCCCTCTCCTGGATTGCACAGCACGATTGAGGGTATGCCACCTTTAATTTGAACTGGCTTGCCGTACTTACAGTTTGATTGCCAGTCCCTTTGGGCCCCAATAAGCTC
TTTCCAGTGCTTTAGCTTTAGATAATGCGGAGCTATGTCATCAATGACGTTATACTCCGCATTATTTGAAAAGACCTTTGAATTAAAGTCGAGGTGCCCACTCAAATAATTATGTGGTCCTAAAGCACGCGCCCACATGGTCTTGCCGGTTCGTGAATCACCTTCAACTATGATACTA
ATAGGTCTTTCCGCCCGCGCAGCGGCACTCCGACCAAAATAGTCATCTGCCCATGAACTCATCTCGTCCGGGACGTTAGTAAAGGAGGAGAGTTGAAACGGAGGAGCCCATGGTTCCGGAGCCTTAGTAAAGAGTCGCTCTATGTTAGCTCTAACATTATGATAACTAACAATGAACGT
CTTTGGATCTCCAGCCCTTATAATTGCGAGAGCCTCTTCCACACATCCCGCATTGACGGCGTTGTGGTAGACGTCGTCTTTATTTGCCTTTGTACCCCCAGACACCTTGTACTGCCCGGATTCACAATAATCACCATCTTTGGTGATGTAATTCTTGACGGCATTGGTGTCTTTGGCTG
CCTGAATGTTTGGGTGAAAATTGGCAGACCTTCTGGGGTGAGTGATGTCGAAAAATCTAGCATCCTTGATGTTCGACTTTCCTGATAGTTGGATGAGACAGTGTAAATGGGGGAACCCGTCTGAATGTTCCTCTCTTGCGACTCTGATGTATGTGGGTTTGACGACTGACCACGACAGG
GTTTGAAGCATCTGAAGAGCTTCATCTTTGGGTATGTCGCACTGGGGATATGTTAAGAATATATTTCGGGC

CaLCuV-Core

>Cb35SPro
TGCTAAACGAAACGATTTAGGGTTTCGTGGCATTTTTGTAAATAATATTTAGGACTCCAGGGGAGGACGCCAGGAGAGCTCTCTCTAAAACCTATTGTTTTTGGTGTCTTGGTGTCCAATATATACTAAAAGCCTCTGGGGAACACCAGGGGCAAAAGCGGCCATCCGCAATAATATTA
CCGGATGGCCGCGATTTTTTTGGTGTCTTGCGTGGGACCACGCATTAAATGAAATCTAACCAATCACATGCCGCCTGACAAGGTTAGCTATTAAGGCTAACTGAGTGCGCTGTGGGCCTATATAAAGACATGTGATTTCAATTTATGCTTTACTTCGAAAaGCCTAAGCGGCaAGCGGT
ACCCTCGAGCCTCTCCAAATGAAATGAACTTCCTTATATAGAGGAAGGGTCTTGCGAAGGATAGTGGGATTGTGCGTCATCCCTTACGTCAGTGGAGATCTAGAGGATCCAGATCTGAGCTCGCTAGCCTAATCCTGTGTCTGCGACATTAAAAATTCGGATCTATTTTTACGATTCGA
TAACAAATTAATAAATTTTGAAATTTATTACATGATTCTCGTGAACATGAGTTACATAGGATCTGTCCGTTGCGAAACGAACAGCTCTAATTACATGATTAATACCAATAACACCTAGGTTATCTAAATGAGACATTACAAGGCATTTGAATCTACTTAAATATGTCGGCCCAGAAGCT
CTCATCGAAGTCGTCCAGACTTGGAAGTTGAAGTAGGCTTTGCGGAGATCCAATGCTTTCCGTAGGTTGTGGTTGAACCGGACTTGGATGTGGTAGATCCTGGTTCTGGTGTACAACGGGTCCTCTACGCGTTGTATCCTGAAATATAGGGGATTTGGAACCTCCCAGATAAAAACGGA
ATTCTCTGCCTGAGCTACAGTGATGCTCTCCCCGGTGCGTGAATCCATTATCTGCGCAATTGATGTGGAGGAAGATAGAACACCCGCAGTTCAAATCAATGCGTCTTCGGCGTACAGCTCTCCTTTTAGCAATCTTGTGCTGTGCTTTGATAGAGGGGGGCTTCAAGAGTGATGAATTT
TGCATTTTTGGTAGTCCACGCTCTTAGTGATGCATTTTCCTCTTTGTTGAGGAAACTTATATAACTGCTCCCCTCTCCTGGATTGCACAGCACGATTGAGGGTATGCCACCTTTAATTTGAACTGGCTTGCCGTACTTACAGTTTGATTGCCAGTCCCTTTGGGCCCCAATAAGCTCTT
TCCAGTGCTTTAGCTTTAGATAATGCGGAGCTATGTCATCAATGACGTTATACTCCGCATTATTTGAAAAGACCTTTGAATTAAAGTCGAGGTGCCCACTCAAATAATTATGTGGTCCTAAAGCACGCGCCCACATGGTCTTGCCGGTTCGTGAATCACCTTCAACTATGATACTAATA
GGTCTTTCCGCCCGCGCAGCGGCACTCCGACCAAAATAGTCATCTGCCCATGAACTCATCTCGTCCGGGACGTTAGTAAAGGAGGAGAGTTGAAACGGAGGAGCCCATGGTTCCGGAGCCTTAGTAAAGAGTCGCTCTATGTTAGCTCTAACATTATGATAACTAACAATGAACGTCTT
TGGATCTCCAGCCCTTATAATTGCGAGAGCCTCTTCCACACATCCCGCATTGACGGCGTTGTGGTAGACGTCGTCTTTATTTGCCTTTGTACCCCCAGACACCTTGTACTGCCCGGATTCACAATAATCACCATCTTTGGTGATGTAATTCTTGACGGCATTGGTGTCTTTGGCTGCCT
GAATGTTTGGGTGAAAATTGGCAGACCTTCTGGGGTGAGTGATGTCGAAAAATCTAGCATCCTTGATGTTCGACTTTCCTGATAGTTGGATGAGACAGTGTAAATGGGGGAACCCGTCTGAATGTTCCTCTCTTGCGACTCTGATGTATGTGGGTTTGACGACTGACCACGACAGGGTT
TGAAGCATCTGAAGAGCTTCATCTTTGGGTATGTCGCACTGGGGATATGTTAAGAATATATTTCGGGC

CaLCuV-ProFL

>Cb35SEnhPro
TGCTAAACGAAACGATTTAGGGTTTCGTGGCATTTTTGTAAATAATATTTAGGACTCCAGGGGAGGACGCCAGGAGAGCTCTCTCTAAAACCTATTGTTTTTGGTGTCTTGGTGTCCAATATATACTAAAAGCCTCTGGGGAACACCAGGGGCAAAAGCGGCCATCCGCAATAATATTA
CCGGATGGCCGCGATTTTTTTGGTGTCTTGCGTGGGACCACGCATTAAATGAAATCTAACCAATCACATGCCGCCTGACAAGGTTAGCTATTAAGGCTAACTGAGTGCGCTGTGGGCCTATATAAAGACATGTGATTTCAATTTATGCTTTACTTCGAAAaGCCTAAGCGGCaAGCGGT
ACCCTCGAGCCTCTCCAAATGAAATGAACTTCCTTATATAGAGGAAGGGTCTTGCGAAGGATAGTGGGATTGTGCGTCATCCCTTACGTCAGTGGAGATATCACATCAATCCACTTGCTTTGAAGACAGTGGTTGGAACGTCTTCTTTTTCCACGATGCTCCTCGTGGGTGGGGGTCCA
TCTTTGGGACCACTGTCGGCAGAGGCATCTTCAACGATGGCCTTTCCTTTATCGCAATGATGGCATTTGTAGGAGCCACCTTCCTTTTCCACTATCTTCACAATAAAGTGACAGATAGCTGGGCAATGGAATCCGAGGAGGTTTCCGGATATTACCCTTTGTTGAAAAGTCTCAATTGC
CCTTTGGTCTTCTAGAGGATCCAGATCTGAGCTCGCTAGCCTAATCCTGTGTCTGCGACATTAAAAATTCGGATCTATTTTTACGATTCGATAACAAATTAATAAATTTTGAAATTTATTACATGATTCTCGTGAACATGAGTTACATAGGATCTGTCCGTTGCGAAACGAACAGCTCT
AATTACATGATTAATACCAATAACACCTAGGTTATCTAAATGAGACATTACAAGGCATTTGAATCTACTTAAATATGTCGGCCCAGAAGCTCTCATCGAAGTCGTCCAGACTTGGAAGTTGAAGTAGGCTTTGCGGAGATCCAATGCTTTCCGTAGGTTGTGGTTGAACCGGACTTGGA
TGTGGTAGATCCTGGTTCTGGTGTACAACGGGTCCTCTACGCGTTGTATCCTGAAATATAGGGGATTTGGAACCTCCCAGATAAAAACGGAATTCTCTGCCTGAGCTACAGTGATGCTCTCCCCGGTGCGTGAATCCATTATCTGCGCAATTGATGTGGAGGAAGATAGAACACCCGCA
GTTCAAATCAATGCGTCTTCGGCGTACAGCTCTCCTTTTAGCAATCTTGTGCTGTGCTTTGATAGAGGGGGGCTTCAAGAGTGATGAATTTTGCATTTTTGGTAGTCCACGCTCTTAGTGATGCATTTTCCTCTTTGTTGAGGAAACTTATATAACTGCTCCCCTCTCCTGGATTGCAC
AGCACGATTGAGGGTATGCCACCTTTAATTTGAACTGGCTTGCCGTACTTACAGTTTGATTGCCAGTCCCTTTGGGCCCCAATAAGCTCTTTCCAGTGCTTTAGCTTTAGATAATGCGGAGCTATGTCATCAATGACGTTATACTCCGCATTATTTGAAAAGACCTTTGAATTAAAGTC
GAGGTGCCCACTCAAATAATTATGTGGTCCTAAAGCACGCGCCCACATGGTCTTGCCGGTTCGTGAATCACCTTCAACTATGATACTAATAGGTCTTTCCGCCCGCGCAGCGGCACTCCGACCAAAATAGTCATCTGCCCATGAACTCATCTCGTCCGGGACGTTAGTAAAGGAGGAGA
GTTGAAACGGAGGAGCCCATGGTTCCGGAGCCTTAGTAAAGAGTCGCTCTATGTTAGCTCTAACATTATGATAACTAACAATGAACGTCTTTGGATCTCCAGCCCTTATAATTGCGAGAGCCTCTTCCACACATCCCGCATTGACGGCGTTGTGGTAGACGTCGTCTTTATTTGCCTTT
GTACCCCCAGACACCTTGTACTGCCCGGATTCACAATAATCACCATCTTTGGTGATGTAATTCTTGACGGCATTGGTGTCTTTGGCTGCCTGAATGTTTGGGTGAAAATTGGCAGACCTTCTGGGGTGAGTGATGTCGAAAAATCTAGCATCCTTGATGTTCGACTTTCCTGATAGTTG
GATGAGACAGTGTAAATGGGGGAACCCGTCTGAATGTTCCTCTCTTGCGACTCTGATGTATGTGGGTTTGACGACTGACCACGACAGGGTTTGAAGCATCTGAAGAGCTTCATCTTTGGGTATGTCGCACTGGGGATATGTTAAGAATATATTTCGGGC

L2 T-DNA

>L2_TDNA
TGGCAGGATATATTGTGGTGTAAACAAATTGACGCTTAGACAACTTAATAACACATTGCGGACGTTTTTAATGTACTGGGGTGGTTTTTCTTTTCACCAGTGAGACGGGCAACAGCTGATTGCCCTTCACCGCCTGGCCCTGAGAGAGTTGCAGCAAGCGGTCCACGCTGGTTTGCCCC
AGCAGGCGAAAATCCTGTTTGATGGTGGTTCCGAAATCGGCAAAATCCCTTATAAATCAAAAGAATAGCCCGAGATAGGGTTGAGTGTTGTTCCAGTTTGGAACAAGAGTCCACTATTAAAGAACGTGGACTCCAACGTCAAAGGGCGAAAAACCGTCTATCAGGGCGATGGCCCACTA
CGTGAACCATCACCCAAATCAAGTTTTTTGGGGTCGAGGTGCCGTAAAGCACTAAATCGGAACCCTAAAGGGAGCCCCCGATTTAGAGCTTGACGGGGAAAGCCGGCGAACGTGGCGAGAAAGGAAGGGAAGAAAGCGAAAGGAGCGGGCGCCATTCAGGCTGCGCAACTGTTGGGAAG
GGCGATCGGTGTGGGCCTCTTCGCTATTACGCAGCTGGCGAAAGGGGGATGTGCTGCAAGGCGATTAAGTTGGGTAACGCCAGGGTTTTCCCAGTCACGACGTTGTAAAACGACGGCCAGTGAATTCGAGCTCGGTACCCGGGGATCCTCTAGAGACGAGGATGCAAGCTTGCATGCCT
GCAGGTCGATCCCCCCTCAGAAGACCAGAGGGCTATTGAGACTTTTCAACAAAGGGTAATATCGGGAAACCTCCTCGGATTCCATTGCCCAGCTATCTGTCACTTCATCGAAAGGACAGTAGAAAAGGAAGGTGGCTCCTACAAATGCCATCATTGCGATAAAGGAAAGGCTATCGTTC
AAGATGCCTCTACCGACAGTGGTCCCAAAGATGGACCCCCACCCACGAGGAACATCGTGGAAAAAGAAGACGTTCCAACCACGTCTTCAAAGCAAGTGGATTGATGTGATGGCCGCTCTAGCATGGAGTCAAAGATTCAAATAGAGGACCTAACAGAACTCGCCGTAAAGACTGGCGAA
CAGTTCATACAGAGTCTCTTACGACTCAATGACAAGAAGAAAATCTTCGTCAACATGGTGGAGCACGACACACTTGTCTACTCCAAAAATATCAAAGATACAGTCTCAGAAGACCAAAGGGCAATTGAGACTTTTCAACAAAGGGTAATATCCGGAAACCTCCTCGGATTCCATTGCCC
AGCTATCTGTCACTTTATTGTGAAGATAGTGGAAAAGGAAGGTGGCTCCTACAAATGCCATCATTGCGATAAAGGAAAGGCCATCGTTGAAGATGCCTCTGCCGACAGTGGTCCCAAAGATGGACCCCCACCCACGAGGAGCATCGTGGAAAAAGAAGACGTTCCAACCACGTCTTCA
AAGCAAGTGGATTGATGTGATATCTCCACTGACGTAAGGGATGACGCACAATCCCACTATCCTTCGCAAGACCCTTCCTCTATATAAGGAAGTTCATTTCATTTGGAGAGGACAGGCTTCTTGAGATCCTTCAACAATTACCAACAACAACAAACAACAAACAACATTACAATTACTAT
TTACAATTACACCATGGGTAAGGGAGAGGAGCTCTTCACCGGGGTGGTGCCCATCCTGGTCGAGCTGGACGGCGACGTAAACGGCCACAAGTTCAGCGTGTCCGGCGAGGGCGAGGGCGATGCCACCTACGGCAAGCTGACCCTGAAGTTCATCTGCACCACCGGCAAGCTGCCCGTGC
CCTGGCCCACCCTCGTGACCACCTTCAGCTACGGCGTGCAGTGCTTCAGCCGCTACCCCGACCACATGAAGCAGCACGACTTCTTCAAGTCCGCCATGCCCGAAGGCTACGTCCAGGAGCGCACCATCTTCTTCAAGGACGACGGCAACTACAAGACCCGCGCCGAGGTGAAGTTCGAG
GGCGACACCCTGGTGAACCGCATCGAGCTGAAGGGCATCGACTTCAAGGAGGACGGCAACATCCTGGGGCACAAGCTGGAGTACAACTACAACAGCCACAACGTCTATATCATGGCCGACAAGCAGAAGAACGGCATCAAGGTGAACTTCAAGATCCGCCACAACATCGAGGACGGCAG
CGTGCAGCTCGCCGACCACTACCAGCAGAACACCCCCATCGGCGACGGCCCCGTGCTGCTGCCCGACAACCACTACCTGAGCACCCAGTCCGCCCTGAGCAAAGACCCCAACGAGAAGCGCGATCACATGGTCCTGCTGGAGTTCGTGACCGCCGCCGGGATCACTCACGGCATGGATG
AACTATACAAATAAAGGATCCTCTAGAGTCCGCAAAAATCACCAGTCTCTCTCTACAAATCTATCTCTCTCTATTTTTCTCCAGAATAATGTGTGAGTAGTTCCCAGATAAGGGAATTAGGGTTCTTATAGGGTTTCGCTCATGTGTTGAGCATATAAGAAACCCTTAGTATGTATTTG
TATTTGTAAAATACTTCTATCAATAAAATTTCTAATTCCTAAAACCAAAATCCAGTGACCTGCAGGCATGCAAGCTTGGCGTAATCATGGTCATAGCTGTTTCCTGTGTGAAATTGTTATCCGCTCACAATTCCACACAACATACGAGCCGGAAGCATAAAGTGTAAAGCCTGGGGTGC
CTAATGAGTGAGCTAACTCACATTAATTGCGTTGCGCTCACTGCCCGCTTTCCAGTCGGGAAACCTGTCGTGCCAGCTGCATTAATGAATCGGCCAACGCGCGGGGAGAGGCGGTTTGCGTATTGGGCCAAAGACAAAAGGGCGACATTCAACCGATTGAGGGAGGGAAGGTAAATATT
GACGGAAATTATTCATTAAAGGTGAATTATCACCGTCACCGACTTGAGCCATTTGGGAATTAGAGCCAGCAAAATCACCAGTAGCACCATTACCATTAGCAAGGCCGGAAACGTCACCAATGAAACCATCGATAGCAGCACCGTAATCAGTAGCGACAGAATCAAGTTTGCCTTTAGCG
TCAGACTGTAGCGCGTTTTCATCGGCATTTTCGGTCATAGCCCCCTTATTAGCGTTTGCCATCTTTTCATAATCAAAATCACCGGAACCAGAGCCACCACCGGAACCGCCTCCCTCAGAGCCGCCACCCTCAGAACCGCCACCCTCAGAGCCACCACCCTCAGAGCCGCCACCAGAACC
ACCACCAGAGCCGCCGCCAGCATTGACAGGAGGCCCGATCTAGTAACATAGATGACACCGCGCGCGATAATTTATCCTAGTTTGCGCGCTATATTTTGTTTTCTATCGCGTATTAAATGTATAATTGCGGGACTCTAATCATAAAAACCCATCTCATAAATAACGTCATGCATTACATG
TTAATTATTACATGCTTAACGTAATTCAACAGAAATTATATGATAATCATCGCAAGACCGGCAACAGGATTCAATCTTAAGAAACTTTATTGCCAAATGTTTGAACGATCGGGGATCATCCGGGTCTGTGGCGGGAACTCCACGAAAATATCCGAACGCAGCAAGATATCGCGGTGCAT
CTCGGTCTTGCCTGGGCAGTCGCCGCCGACGCCGTTGATGTGGACGCCGGGCCCGATCATATTGTCGCTCAGGATCGTGGCGTTGTGCTTGTCGGCCGTTGCTGTCGTAATGATATCGGCACCTTCGACCGCCTGTTCCGCAGAGATCCCGTGGGCGAAGAACTCCAGCATGAGATCCC
CGCGCTGGAGGATCATCCAGCCGGCGTCCCGGAAAACGATTCCGAAGCCCAACCTTTCATAGAAGGCGGCGGTGGAATCGAAATCTCGTGATGGCAGGTTGGGCGTCGCTTGGTCGGTCATTTCGAACCCCAGAGTCCCGCTCAGAAGAACTCGTCAAGAAGGCGATAGAAGGCGATGC
GCTGCGAATCGGGAGCGGCGATACCGTAAAGCACGAGGAAGCGGTCAGCCCATTCGCCGCCAAGCTCTTCAGCAATATCACGGGTAGCCAACGCTATGTCCTGATAGCGGTCCGCCACACCCAGCCGGCCACAGTCGATGAATCCAGAAAAGCGGCCATTTTCCACCATGATATTCGGC
AAGCAGGCATCGCCATGGGTCACGACGAGATCATCGCCGTCGGGCATGCGCGCCTTGAGCCTGGCGAACAGTTCGGCTGGCGCGAGCCCCTGATGCTCTTCGTCCAGATCATCCTGATCGACAAGACCGGCTTCCATCCGAGTACGTGCTCGCTCGATGCGATGTTTCGCTTGGTGGTC
GAATGGGCAGGTAGCCGGATCAAGCGTATGCAGCCGCCGCATTGCATCAGCCATGATGGATACTTTCTCGGCAGGAGCAAGGTGAGATGACAGGAGATCCTGCCCCGGCACTTCGCCCAATAGCAGCCAGTCCCTTCCCGCTTCAGTGACAACGTCGAGCACAGCTGCGCAAGGAACGC
CCGTCGTGGCCAGCCACGATAGCCGCGCTGCCTCGTCCTGCAGTTCATTCAGGGCACCGGACAGGTCGGTCTTGACAAAAAGAACCGGGCGCCCCTGCGCTGACAGCCGGAACACGGCGGCATCAGAGCAGCCGATTGTCTGTTGTGCCCAGTCATAGCCGAATAGCCTCTCCACCCAA
GCGGCCGGAGAACCTGCGTGCAATCCATCTTGTTCAATCATGCGAAACGATCCAGATCCGGTGCAGATTATTTGGATTGAGAGTGAATATGAGACTCTAATTGGATACCGAGGGGAATTTATGGAACGTCAGTGGAGCATTTTTGACAAGAAATATTTGCTAGCTGATAGTGACCTTAG
GCGACTTTTGAACGCGCAATAATGGTTTCTGACGTATGTGCTTAGCTCATTAAACTCCAGAAACCCGCGGCTGAGTGGCTCCTTCAACGTTGCGGTTCTGTCAGTTCCAAACGTAAAACGGCTTGTCCCGCGTCATCGGCGGGGGTCATAACGTGACTCCCTTAATTCTCCGCTCATGA
TCAGATTGTCGTTTCCCGCCTTCAGTTTAAACTATCAGTGTTTGACAGGATATATTGGCGGGTAAACTGGCAGGATATATTGTGGTGTAAACAAATTGACGCTTAGACAACTTAATA

L2 Lead

>L2_Lead
CTTCAACAATTACCAACAACAACAAACAAC

L2 CodM

>L2_midGFP
CGCATCGAGCTGAAGGGCATCGACTTCAAG

L2 Trail

>L2_Term
TCCGCAAAAATCACCAGTCTCTCTCTACAA

L2 PolyA

>L2_PolyA
TTCTATCAATAAAATTTCTAATTCCTAAAA

L2 Enh

>L2_Enh
AGAAGACCAAAGGGCAATTGAGACTTTTCAACAAAGGGTAATATCCGGAAACCTCCTCGGATTCCATTGCCCAGCTATCTGTCACTTTATTGTGAAGATAGTGGAAAAGGAAGGTGGCTCCTACAAATGCCATCATTGCGATAAAGGAAAGGCCATCGTTGAAGATGCCTCTGCCGACA
GTGGTCCCAAAGATGGACCCCCACCCACGAGGAGCATCGTGGAAAAAGAAGACGTTCCAACCACTGTCTTCAAAGCAAGTGGATTGATGTGATA

L2 ProFL

>L2_EnhPro

AGAAGACCAAAGGGCAATTGAGACTTTTCAACAAAGGGTAATATCCGGAAACCTCCTCGGATTCCATTGCCCAGCTATCTGTCACTTTATTGTGAAGATAGTGGAAAAGGAAGGTGGCTCCTACAAATGCCATCATTGCGATAAAGGAAAGGCCATCGTTGAAGATGCCTCTGCCGACA
GTGGTCCCAAAGATGGACCCCCACCCACGAGGAGCATCGTGGAAAAAGAAGACGTTCCAACCACTGTCTTCAAAGCAAGTGGATTGATGTGATATCTCCACTGACGTAAGGGATGACGCACAATCCCACTATCCTTCGCAAGACCCTTCCTCTATATAAGGAAGTTCATTTCATTTGGA
GAGG

L2 Core

>L2_Pro
TCTCCACTGACGTAAGGGATGACGCACAATCCCACTATCCTTCGCAAGACCCTTCCTCTATATAAGGAAGTTCATTTCATTTGGAGAGG
